# Pregnane X receptor deletion induces a lean phenotype in chow-fed mice through a liver–ileum–muscle axis involving 6α-hydroxylated bile acids and Fgf15

**DOI:** 10.1016/j.molmet.2026.102442

**Published:** 2026-09-19

**Authors:** Bérengère Benoit, Audrey Jalabert, Emmanuelle Meugnier, Stéphanie Chanon, Aurélie Vieille-Marchiset, Alice Beau, Sandra Pesenti, Emmanuelle Loizon, Oriane Vitalis, Nadia Bendridi, Margaux Nawrot, Kassem Makki, Brigitte Le Magueresse-Battistoni, Jennifer Rieusset, Marie-Caroline Michalski, Dominique Rainteau, Karim Chikh, Hubert Vidal, Jérôme Ruzzin

**Affiliations:** 1 Université Lyon1, CarMeN Laboratory, INSERM, INRAE, Pierre Bénite, France; 2 Metanutribiota platform, Centre de Biologie et de Pathologie, Hospices Civils de Lyon, Groupement Hospitalier Sud, Pierre Bénite, France; 3 Sorbonne Université, Saint Antoine Research Center, INSERM UMR 938, Département de Métabolomique Clinique, Hôpital Saint Antoine, AP-HP Sorbonne Université, Paris, France; 4 Department of Biology, University of Bergen, Bergen, Norway; 5 Department of Molecular Medicine, Institute of Basic Medical Sciences, Faculty of Medicine, University of Oslo, Oslo, Norway

**Keywords:** Pregnane X receptor (PXR/NR1I2), Bile acids, FGF15/19, Skeletal muscle, Energy metabolism

## Abstract

**Objectives:**

The pregnane X receptor (Pxr; *Nr1i2*) plays a role in metabolic regulation. However, its contribution to systemic energy homeostasis under physiological conditions remains unclear. Here, we investigated the metabolic consequences of *Nr1i2* deficiency in chow-fed mice.

**Methods:**

Phenotyping was performed in adult whole-body *Nr1i2* knockout (*Nr1i2*^*−/−*^) chow-fed mice and their wild-type littermates. Body composition, metabolic parameters, skeletal muscle mass and myofiber size were assessed. Ileal gene expression, circulating Fgf15 levels, and bile acid composition were analyzed. Intestinal-specific *Nr1i2*^*−/−*^ mice were generated to evaluate the contribution of intestinal Pxr signaling. Mechanistic studies were conducted in HT29 cells and mouse ileal organoids.

**Results:**

Chow-fed *Nr1i2*^*−/−*^ mice displayed a lean phenotype with reduced adiposity, improved insulin sensitivity, and increased skeletal muscle mass and myofiber size compared with wild-type mice. This phenotype was associated with strong induction of ileal *Fgf15* gene expression and elevated circulating Fgf15 levels. Intestinal-specific *Nr1i2* deletion did not reproduce the metabolic or muscular phenotype, indicating that intestinal Pxr loss alone is insufficient. *Nr1i2* deficiency was associated with remodeling of the hepatic bile acid pathway and enrichment of specific taurine-conjugated and 6α-hydroxylated bile acids in the ileum. *In vitro*, these bile acids enhanced FGF19 expression through FXR-related canonical and non-canonical mechanisms, supporting a mechanistic link between bile acid remodeling and increased Fgf15/19 signaling.

**Conclusions:**

These findings provide the first evidence that, under standard dietary conditions, loss of Pxr induces a lean phenotype, promotes reduced adiposity together with remodeling of bile acid pool, increased Fgf15 production and enhanced skeletal muscle mass.

## HIGHLIGHTS

• Loss of *Nr1i2* causes a lean phenotype under standard chow feeding.
• Loss of *Nr1i2* increases skeletal muscle mass and myofiber size.
• This phenotype is associated with increased ileal/circulating Fgf15 signaling.
• Intestinal *Nr1i2* loss alone is insufficient to recapitulate the phenotype.
• Bile acid remodeling is a plausible upstream mechanism.

## Graphical abstract

**Figure:**
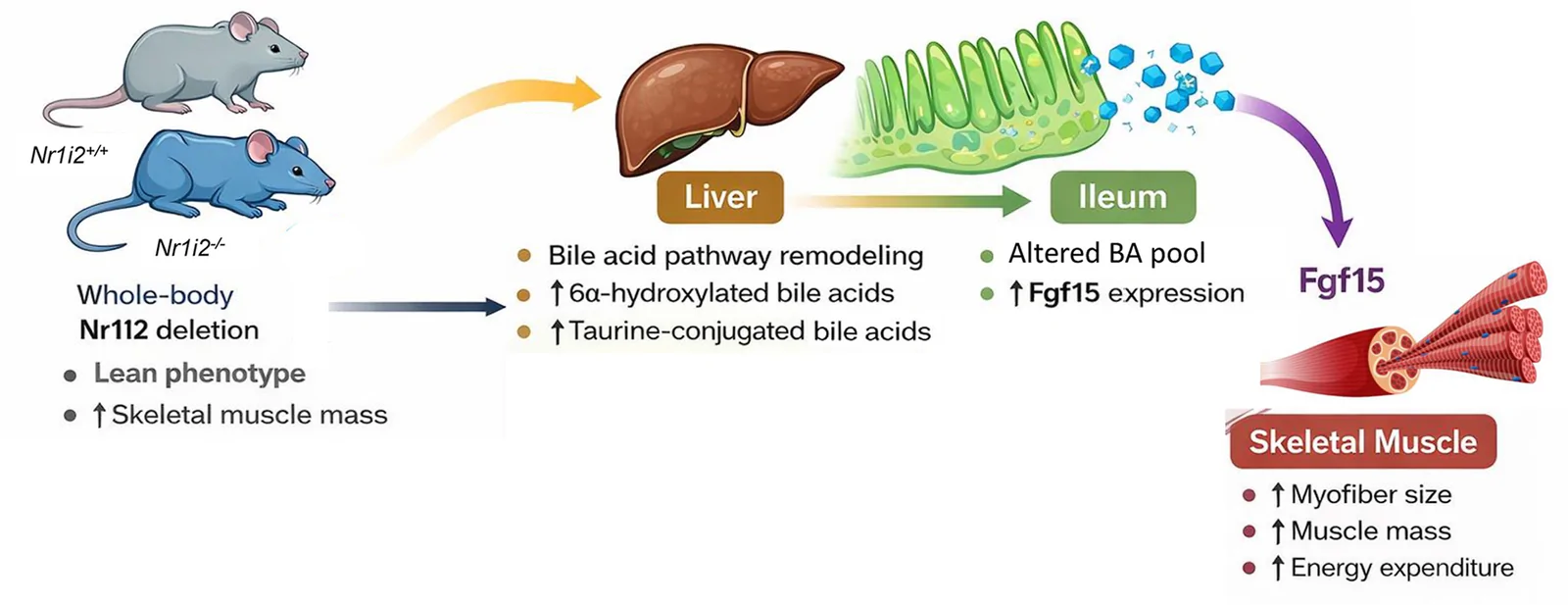

## 1 Introduction

The pregnane X receptor (Pxr), encoded by the *Nr1i2* gene, is a nuclear receptor predominantly expressed in the liver and small intestine. It regulates the transcription of phase I-III genes involved in the metabolism, transport, and excretion of both endobiotics and xenobiotics [[1], [2], [3], [4]]. Beyond its canonical role in detoxification, accumulating evidence indicates that Pxr also contributes to the regulation of energy metabolism. Several studies have highlighted the metabolic consequences of *Nr1i2* deficiency under conditions of metabolic stress. For example, when challenged with a high-fat diet (HFD), *Nr1i2*-deficient (*Nr1i2*^*−/−*^) mice were protected from obesity and maintained insulin sensitivity [5]. Similarly, *ob/ob* mice lacking Pxr showed enhanced energy expenditure, increased lean body mass and an improved diabetic phenotype [5]. Mechanistically, Pxr deficiency has been associated with reduced hepatic lipogenesis, increased mitochondrial β-oxidation, and restored hepatic insulin signaling in HFD-fed mice [5]. Consistently, Spruiell et al. found an upregulation of the lipid sensor Pparα in the liver of Pxr deficient mice, suggesting a metabolic shift toward enhanced energy expenditure [6]. Conversely, pharmacological activation of Pxr has been shown to promote hepatic lipid accumulation in mice [7].

Pxr also plays an important role in the regulation of bile acid metabolism and composition [8]. Beyond their classical role in lipid absorption, bile acids act as endocrine regulators of whole-body metabolism. Specific bile acids, such as cholic acid (CA) and ursodeoxycholic acid (UDCA), counteract HFD-induced obesity in mice [[9], [10], [11]]. More recently, 6α-hydroxylated secondary bile acids have been shown to improve glucose homeostasis in rodent models [12]. The beneficial effects of bile acids may involve increased energy expenditure and brown adipose tissue thermogenesis [13], as well as the stimulation of enteroendocrine hormone secretion [12,14,15], including glucagon-like peptide 1 (GLP-1) and fibroblast growth factor (FGF) 15/19.

FGF15/19 (Fgf15 in mice and FGF19 in humans) emerges as a central regulator of metabolic homeostasis. Secreted by ileal enterocytes in response to bile acids, FGF15/19 classically inhibits hepatic CYP7A1, the rate-limiting enzyme in bile acid synthesis [16]. In preclinical models, including HFD-fed and *ob/ob* mice, FGF15/19 promotes energy expenditure and protects against diabetes and metabolic dysfunction [17,18]. Recent studies, including our own, further demonstrated that FGF19 directly promotes skeletal muscle hypertrophy, thereby contributing to increased muscle mass and improved whole-body metabolic function [[19], [20], [21]]. These observations raise the possibility that Pxr signaling may influence not only adiposity and glucose homeostasis, but also skeletal muscle mass.

Despite these advances, most studies investigating the metabolic consequences of Pxr deficiency have focused on models of diet-induced or genetic obesity. Whether *Nr1i2* deletion influences whole-body homeostasis under physiological dietary conditions remains poorly understood. Therefore, we investigated whether *Nr1i2* deletion induces a distinct metabolic phenotype under standard chow feeding, with particular attention to a possible involvement of bile acid remodeling and Fgf15 in this response.

## 2 Materials and methods

### 2.1 Whole-body deletion of *Nr1i2* (*Nr1i2*^*+/+*^ and *Nr1i2*^*−/−*^ male mice)

Male *Nr1i2*^+/+^ and *Nr1i2*^−/−^ mice [3], generated by targeted deletion of the first coding exon encoding the PXR DNA-binding domain, were maintained on a 129S6/SvEvTac genetic background (gift from RM Evans, California, USA). Mice were housed in a ventilated system (4–6 per cage) at controlled temperature (22–23 °C) with ad libitum access to food. All animal experiments were approved by the Norwegian State Board of Biological Experiments with Living Animals and conducted and reported in accordance with ARRIVE 2.0 guidelines. Independent studies were performed in mice fed either a chow diet (7.5% of calories from fat, 17.5% from protein, and 75% carbohydrates; product code 80 1151, Special Diets Services, England) for 22 weeks or 17 months, or HFD (D12492 without dye, Brogaarden, Denmark: 60% fat, 20% protein, 20% carbohydrates) for 16 weeks. *Nr1i2*^*+/+*^ (control) and *Nr1i2*^*−/−*^ mice were allocated to experimental groups according to genotype and diet and otherwise handled identically. Each animal represented one experimental unit. Body weight and food intake were recorded throughout the study. At completion, mice were fasted for 4 h, anaesthetized with isoflurane and euthanized by cardiac puncture. Blood was collected in EDTA-coated tubes and tissues were dissected, weighed, and either snap-frozen in liquid nitrogen for gene expression and bile acid analyses or fixed for immunohistochemistry.

### 2.2 Intestinal deletion of *Nr1i2* (*intNr1i2*^*+/+*^ and *intNr1i2*^*−/−*^ male mice)

*Nr1i2*^*fl/fl*^ mice displaying *LoxP* sites flanking exon X of the *Nr1i2* (Mouse Clinical Institute (ID: 7010/K177-27 L2/0231–2/Nr1i2/PXRcKO), Illkirch, France) were crossed with Villin-Cre mice (IGBMC, Illkirch, France) to generate intestinal-specific control (*intNr1i2*^*+/+*^*)* and knockout (*intNr1i2*^*−/−*^*)* mice on a C57BL/6 background. Exon X excision was validated by RT-PCR analysis of *Nr1i2* expression in ileum and liver. Male mice (6 weeks old) were housed in a controlled environment (4–6 per cage) with ad libitum access to food. *IntNr1i2*^*+/+*^ and *intNr1i2*^*−/−*^ mice were allocated to experimental groups according to genotype and otherwise handled identically. All procedures complied with French Ministry of Agriculture guidelines (n°2013–118) and EU Directive 2010/63/EU, were approved by the local ethics committee (APAFIS #2017062215163203-v2) and conducted and reported in accordance with ARRIVE 2.0 guidelines.

Mice were fed a chow diet (17.8 % protein, 10.2 % fat, 72 % carbohydrates; LASQC diet® Rod-16 R, LASvendi) until 22 weeks of age, and body weight was monitored throughout. At completion, mice were fasted for 4 h, anesthetized with isoflurane, and euthanized by cardiac puncture. Blood was collected in EDTA-coated tubes, and tissues were dissected, weighed, and either snap-frozen in liquid nitrogen for gene expression and bile acid analyses or fixed for histology.

### 2.3 Glucose and insulin tolerance tests

Glucose and insulin tolerance tests were performed after a 5-h fast. Mice received intraperitoneal injections of glucose (1.0 g/kg body weight) or human insulin (Actrapid, Novo Nordisk, Denmark; 0.75 U/kg body weight). Blood glucose levels were measured using a glucometer (Accu-Check Performa, Roche Diagnostics, France) at baseline (0 min) and 15, 30, 60, and 90 min post-injection.

### 2.4 Indirect calorimetry and activity

Animals were individually housed in indirect calorimetry chambers (Comprehensive Lab Animal Monitoring System, Columbus Instruments, USA). Following a 24-h acclimation period, oxygen consumption and carbon dioxide production were recorded for 48 h. Physical activity was assessed by ambulatory and stereotypical counts, and rectal temperature was measured using a BAT-12 microprobe thermometer (Physitemp, USA).

### 2.5 Biochemistry

Plasma insulin, triglycerides, and Fgf15 concentrations were measured using ELISA or enzymatic kits (ALPCO Diagnostics, EuroBio Laboratoires, France; ref. 80-INSMSU-E01; Biolabo SAS, France, ref. 80,019, and Antibodies-online GmbH, Germany; ref. ABIN481717, respectively).

Bile acids were quantified in frozen ileum (20–50 mg) and liver (50 mg) samples by high-performance liquid chromatography coupled to tandem mass spectrometry (HPLC-MS/MS), as described [22,23]. Results were expressed as nmol/g wet tissue.

### 2.6 Immunohistochemistry

Mouse skeletal muscles (soleus and tibialis anterior) were embedded in OCT and snap-frozen in liquid nitrogen. Transverse mid-belly cryosections (10 μm) were immunolabeled with an anti-laminin antibody (L9393, Sigma), followed by Alexa Fluor 594–conjugated goat anti-rabbit IgG (A11012, Thermo Fisher). Images were acquired using an AxioCam system (Zeiss, Germany) and analyzed with Automeasure software (Zeiss), as described [19,20]. Only transverse fibers with a Feret ratio ≤0.5 were included, and fiber cross-sectional area was measured in μm^2^.

### *2.7* *Ex-vivo* intestinal experiments

Following anesthesia, small intestines from overnight-fasted mice were isolated, and a 1-cm distal ileum segment was collected, washed, and incubated for 2.5 h at 37 °C in 1 mL high-glucose DMEM supplemented with glutamine, pyruvate, 10 % calf serum, penicillin (100 U/mL), and streptomycin (100 U/mL). *Fgf1*5 mRNA levels were quantified by RT-qPCR.

### *2.8* *In vitro* studies on HT29 cells

The human intestinal cancer cell line HT29 (ATCC) was cultured according to supplier's instructions (DMEM high-glucose, 1% sodium pyruvate, 1% penicillin-streptomycin and 10 % fetal bovine serum, 37 °C, 5% CO2). Cells were seeded in 24-well plates and treated two days after confluence for 16 h in serum-free medium. Experiments were performed in triplicate (n = 3 wells) across 5–7 independent experiments using HDCA (No. 20294), TCA (No. 16215), TDCA (No. 15935), THDCA (No. 21956), TUDCA (No. 20277) or β-TMCA (No. 20289) all at 100 μM, with or without the FXR agonist GW4064 (0.1 μM, No. 10006611). All bile acids were diluted in DMSO and purchased from Cayman Chemicals (Bertin Technologies, France). RNA was extracted using TRizol and supernatants were stored at −80 °C for FGF19 quantification. Secreted FGF19 protein levels were quantified in cell culture supernatants using a human FGF19 Quantikine ELISA kit (R&D Systems, Bio-Techne SAS, USA, ref. DF1900), according to the manufacturer's instructions. Concentrations were calculated from the standard curve and expressed as relative to the control condition.

### *2.9* *In vitro* studies on mouse ileal organoids

Intestinal crypts were isolated from ileum of WT C57BL/6 J mice, embedded in Matrigel® (≈20 crypts per μL, CSL356231 Stem Cell, France) and plated (25 μL per well) in pre-warmed 48-well plates. After polymerization (37 °C, 20 min), IntestiCult™ Organoid Growth Medium (6005, StemCell Technologies, 250μL/well) was added. Plates were incubated at 37 °C with 5% CO_2_ and medium was renewed three times weekly. Organoids were passaged every 5 days (1:2 ratio). Mature organoids (5 days post-passage, ≈130–150 per well) were treated overnight in triplicate (n = 3 wells; 3–4 independent experiments) with DMSO, GW4064 (0.01 μM), THDCA (100 μM), HDCA (100 μM), or combinations of GW4064 with THDCA or HDCA. Total RNA was extracted using the miRneasy Micro kit (Qiagen, France).

### 2.10 Gene expression analysis

Following total RNA extraction, levels of specific mRNAs were quantified using real-time qPCR as described [24]. Briefly, cDNA was synthesized from 1 μg of RNA using the TAKARA Prime Script™ RT kit (TAKARA Bio, France). qPCR was performed with SYBR® Premix Ex-Taq™ on a Rotor-Gene™ Q (Qiagen, France). *TBP* was used as housekeeping gene for all the normalizations of our genes of interest. All primers are listed in Supplementary Tables 1, 2 and 3.

### 2.11 Data analysis and statistics

Statistical analyses and data visualization were performed using GraphPad Prism 8.0 (GraphPad Software), Python (v3.10) and R (v4.3. x). Animal allocation, sample processing, and analyses were performed using predefined standardized protocols; blinding was not applied. To minimize potential confounders, animals from different experimental groups were housed under identical conditions, handled in parallel and analyzed using standardized protocols.

Sample size was determined based on prior experience with this model and on effect sizes observed in comparable studies; no formal a priori power calculation was performed. No a priori inclusion or exclusion criteria were defined at the animal level. Outliers were identified using a predefined objective criterion (values exceeding ±2 standard deviations from the group mean). Exclusions were limited and did not alter the overall conclusions.

Group comparisons were performed using statistical tests appropriate to data distribution. Normally distributed data (assessed using a Shapiro–Wilk test) were analyzed using Student's t-tests or one-way ANOVA, followed by Dunnett's multiple-comparison test. Non-normally distributed data were analyzed using Mann–Whitney U or Kruskal–Wallis tests, followed by Dunn's multiple-comparison test. Body-weight changes were analyzed using a repeated-measures linear mixed-effects model. A 95% confidence interval was applied throughout.

Spearman's rank correlation coefficients (ρ) were used to assess relationships between selected parameters. Pairwise correlations between ileal *Fgf15* expression and hepatic gene expression were corrected for multiple testing using the Benjamini–Hochberg false discovery rate (FDR), with significance defined as *q* < 0.05. These analyses were performed in Python using the *pandas*, *scipy. stats*, and *statsmodels* libraries, and visualized with *matplotlib*.

For cell culture experiments in HT29 cells and mouse ileal organoids, group differences were evaluated using Kruskal–Wallis tests followed by Dunn's post hoc tests.

Statistical details are provided in the figure legends and in Supplementary Material. Results are presented as means ± SEM. Significance thresholds are defined as: *p* ≤ 0.05, *p* ≤ 0.01, *p* ≤ 0.001.

## 3 Results

### *3.1* *Nr1i2*^*−/−*^ mice display a lean phenotype with increased skeletal muscle mass under chow feeding

*Nr1i2* expression was absent in the liver, ileum and soleus skeletal muscle of *Nr1i2*^*−/−*^ mice, whereas robust expression was observed in the liver and ileum of control mice, thereby validating the *Nr1i2*^*−/−*^ mouse model (Supp. Fig. 1).

Under chow feeding, *Nr1i2*^*−/−*^ mice exhibited a lean phenotype (Genotype × Time interaction (p < 0.001), Figure 1A), characterized by reduced WAT mass despite increased food intake (Figure 1A–B). Moreover, fasting insulin and triglyceride levels were significantly reduced in *Nr1i2*^*−/−*^ mice, while fasting glucose showed a non-significant downward trend (p = 0.10; Fig. 1C). Unexpectedly, *Nr1i2*^*−/−*^ mice also exhibited a significant increase in skeletal muscle mass (Fig. 1D). Analysis of soleus and tibialis anterior muscle fiber size distribution using a mixed-effect modeling approach revealed a significant Genotype x Fiber size (bin) interaction (p < 0.0001 and p < 0.01, respectively), indicating a marked shift toward larger fiber sizes in *Nr1i2*^*−/−*^ mice compared with *Nr1i2*^*+/+*^ mice (Figure 1E–F). To further characterize the skeletal muscle phenotype at the molecular level, we measured selected markers related to Fgf15 responsiveness, energy metabolism, glycogen metabolism and muscle remodeling in the soleus muscle. The expression of *Fgfr4* and *Klb*, encoding components of the Fgf15 receptor machinery, was not significantly altered in *Nr1i2*^*−/−*^ mice compared with *Nr1i2*^*+/+*^ mice (Supp. Fig. 2). Similarly, the expression of the atrophy-related genes *Fbxo32* and *Trim63*, as well as *Ucp2*, remained unchanged. In contrast, the expression of *Dio2*, *Ucp3*, *Gys1*, and *Myod1* was significantly increased in the soleus muscle of *Nr1i2*^*−/−*^ mice (Supp. Fig. 2). These changes are consistent with a transcriptional profile suggestive of enhanced oxidative/energy metabolism, increased glycogen storage capacity, and muscle remodeling/myogenic activity.

**Figure 1:**
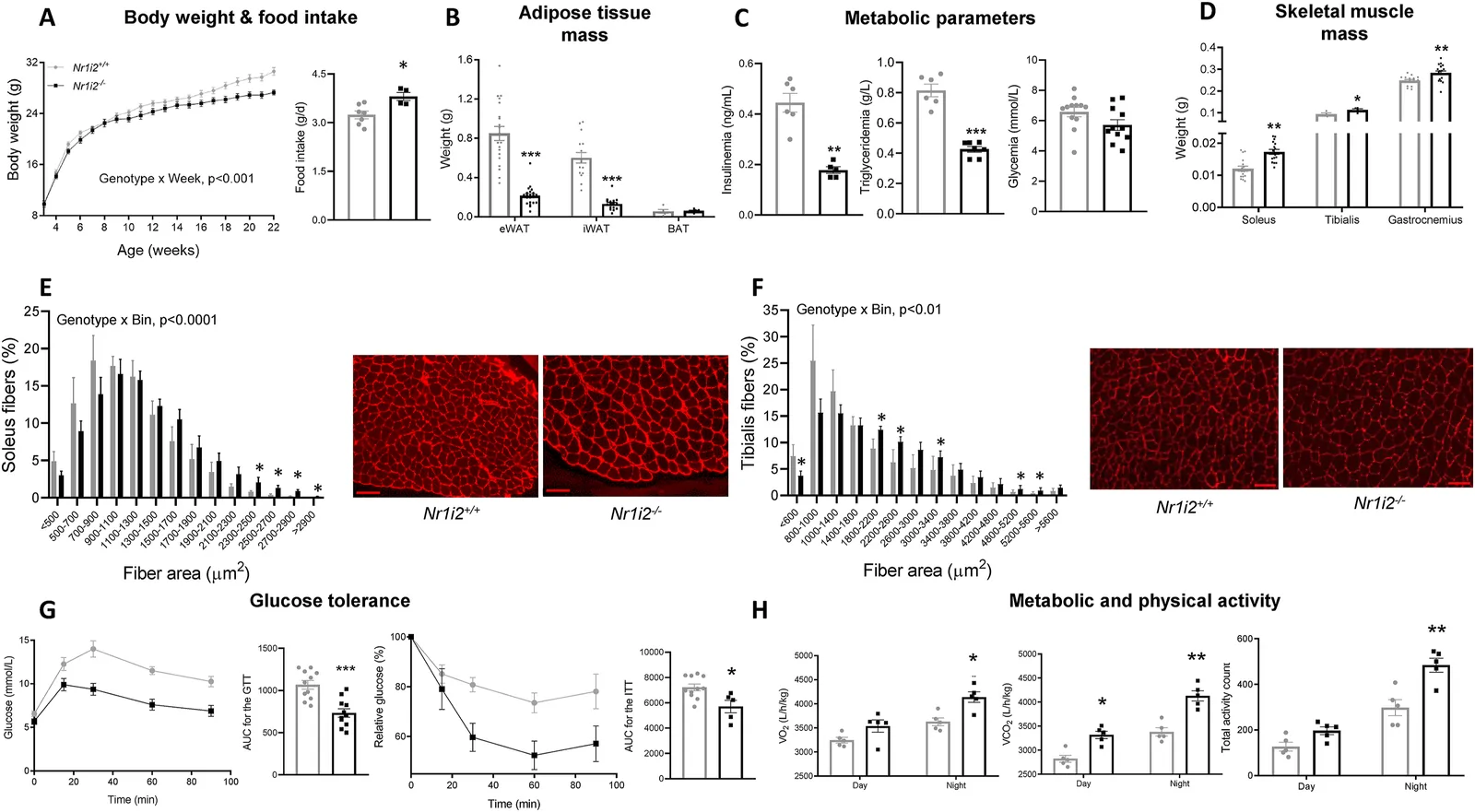
***Nr1i2*^*−/−*^mice display a lean phenotype with improved metabolic parameters and pronounced skeletal muscle hypertrophy.** (A) Longitudinal changes in body weight (g) from birth to 22 weeks of age and food intake (measured during week 14 of the protocol). (B) Weights of epididymal white adipose tissue (eWAT), inguinal white adipose tissue (iWAT), and brown adipose tissue (BAT) collected from 22-week-old mice. (C) Plasma insulin, triglycerides, and blood glucose levels (parameters measured at 22 weeks following 4 h of fasting). (D) Weights of soleus, tibialis anterior, and gastrocnemius muscles collected from 22-week-old mice. Analysis of muscle fiber distribution by size in the soleus (E) and tibialis anterior (F) muscles of *Nr1i2*^*+/+*^ and *Nr1i2*^*−/−*^ mice. (E) Soleus muscle: graphs showing fiber distribution (as %) based on area (in μm^2^) along with microscopic images of soleus muscle cross-sections from *Nr1i2*^*+/+*^ and *Nr1i2*^*−/−*^ mice (laminin stained in red, scale bar = 50 μm). (F) Tibialis anterior muscle: graphs showing fiber distribution (as %) based on area (in μm^2^), along with microscopic images of tibialis anterior muscle cross-sections from *Nr1i2*^*+/+*^ and *Nr1i2*^*−/−*^ mice (laminin stained in red, scale bar = 50 μm). (G) Glucose tolerance test (blood glucose levels (mmol/L) over time (min) and area under the curve (AUC) calculated from the test data) and insulin sensitivity test (changes in blood glucose levels (expressed as a percentage of fasting blood glucose at t0) over time (min), with AUC calculated from the test data). (H) VO_2_ (L/h/kg), VCO_2_ (L/h/kg), and total activity counts measured during week 20, assessed during both the light and dark phases. Data are presented as mean ± SEM. ∗p < 0.05, ∗∗p < 0.01, ∗∗∗p < 0.001. *Nr1i2*^*+/+*^ mice are represented in grey, and *Nr1i2*^*−/−*^ mice in black. Group sizes ranged from n = 5 to 13 mice per group, depending on the parameter analyzed.

Consistent with the overall phenotype associating leanness and increased muscle mass, both glucose and insulin tolerance were significantly improved in *Nr1i2*^*−/−*^ mice (Fig. 1G). Indirect calorimetry demonstrated elevated oxygen consumption during the dark phase and increased CO_2_ production across the entire light–dark cycle in *Nr1i2*^*−/−*^ mice, consistent with a higher basal metabolic rate. In addition, these mice exhibited greater spontaneous locomotor activity, particularly during the dark (active) period, further supporting an overall increase in energy expenditure (Fig. 1H).

Because increased energy expenditure could theoretically reflect enhanced thermogenesis, we assessed BAT activation and WAT browning (Supp. Fig. 3). However, thermogenic gene expression and rectal temperature were globally unchanged, arguing against a major contribution of classical thermogenesis to the phenotype (Supp. Figure 3A–C). Hepatic lipid metabolism was then assessed. mRNA levels of major lipid metabolic regulators (*Srebp1c, Fasn, Pparα, Pgc1α, Pdk4* and *Cpt1a*) were unchanged (Supp. Fig. 3D). Only *Cd36* and *Dgat2* were reduced in *Nr1i2*^*−/−*^ mice, while *Ucp2* was increased in these same mice (Supp. Fig. 3D).

Importantly, this chow-fed phenotype was maintained with aging (Supp. Fig. 4) and remained detectable under HFD challenge (Supp. Fig. 5) as previously reported [5], together indicating that the effects of *Nr1i2* deletion are robust across conditions.

### 3.2 Whole-body *Nr1i2* deletion increases ileal Fgf15 expression and production

We next investigated whether whole-body PXR deletion induced ileal expression of *Fgf15*, a hormone with well-established hypertrophic effects on skeletal muscle [[17], [18], [19]] and previously reported to be overexpressed in HFD-fed *Nr1i2*^−/−^ mice [25]. In 22-week-old chow-fed mice, ileal *Fgf1*5 mRNA levels were significantly elevated in *Nr1i2*^−/−^ mice compared with controls (Figure 2A). Consistently, *ex vivo* analysis revealed that *Fgf1*5 mRNA was undetectable in ileal explants from control mice, whereas it was readily detectable in explants from *Nr1i2*^−/−^ (Fig. 2B), suggesting enhanced Fgf15 production by ileal tissue. Circulating Fgf15 levels were also elevated in *Nr1i2*^*−/−*^ mice (Fig. 2C) and this was associated with marked reduction of hepatic *Cyp7a1* mRNA expression, a well-established target of FGF15/19 signaling [26,27] (Fig. 2D). Together, these findings indicate that systemic *Nr1i2* deletion is associated with enhanced ileal Fgf15 production and increased circulating Fgf15 levels. Since FGF15 primarily acts as an endocrine ileum-derived hormone targeting the liver, hepatic *Cyp7a1* repression is consistent with increased Fgf15 bioactivity *in vivo* under physiological dietary conditions in *Nr1i2*^−/−^ mice.

**Figure 2:**
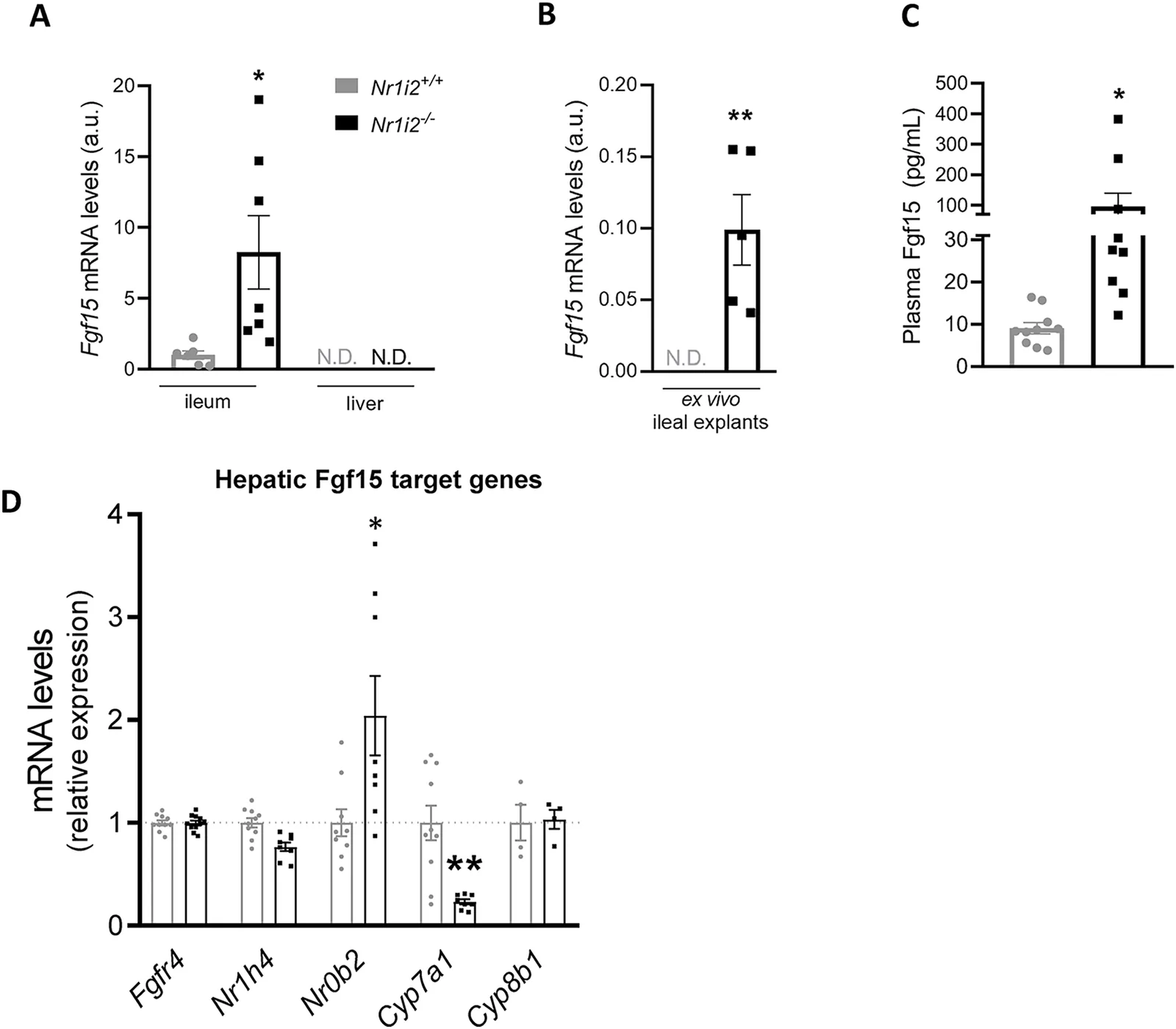
**Whole-body *Nr1i2* deletion increases Fgf15 expression and production in ileum.** (A) *Fgf1*5 mRNA levels measured in the ileum and liver of 22-week-old *Nr1i2*^*+/+*^ and *Nr1i2*^*−/−*^ mice fed a chow diet (a.u. Indicates arbitrary units). (B) *Fgf1*5 mRNA levels measured in ileal explants from *Nr1i2*^*+/+*^ and *Nr1i2*^*−/−*^ mice fed a chow diet, incubated for 2.5 h at 37 °C in high-glucose DMEM culture medium (a.u. Indicates arbitrary units). (C) Plasma concentrations of Fgf15 (pg/mL) measured by ELISA in plasma samples from *Nr1i2*^*+/+*^ and *Nr1i2*^*−/−*^ mice fed a chow diet at 22 weeks of age following a 4-hour fast. (D) Relative gene expression of *Fgfr4, Nr1h4, Nr0b2, Cyp7a1* and *Cyp8b1* in the liver. Gene expression data are presented as fold changes relative to gene expression levels in the control group (*Nr1i2*^*+/+*^ mice). Data are presented as mean ± SEM. ∗p < 0.05, ∗∗p < 0.01. N.D. indicates not detectable. *Nr1i2*^*+/+*^ mice are represented in grey, and *Nr1i2*^*−/−*^ mice in black. Group sizes ranged from n = 5 to 10 mice per group, depending on the parameter analyzed.

### 3.3 Intestinal-specific *Nr1i2* deletion does not recapitulate the phenotype of whole-body *Nr1i2* deficiency

Because Pxr is expressed in the small intestine and Fgf15 produced by the ileum, we investigated whether the ileal Fgf15 induction observed in whole-body knockout mice could be directly driven by intestinal Pxr. To address this question, mice with an intestinal-specific deletion of *Nr1i2* (int*Nr1i2*^*−/−*^) were generated. As expected, *Nr1i2* mRNA expression was completely abolished in the ileum but preserved in the liver of int*Nr1i2*^*−/−*^ mice (Figure 3A). In contrast to whole-body *Nr1i2*^−/−^ mice, int*Nr1i2*^*−/−*^ mice displayed similar body weight gain to wild-type littermates when fed a chow diet for 22 weeks (Fig. 3B). Although a modest increase in ileal *Fgf1*5 mRNA was observed, plasma Fgf15 levels remained unchanged compared with int*Nr1i2*^*+/+*^ mice (Fig. 3C). Consistently, hepatic expression of Fgf15 target genes, including *Cyp7a1*, was unaffected in int*Nr1i2*^*−/−*^ mice (Fig. 3D). Moreover, myofiber size distribution in the tibialis anterior muscle was unchanged (Fig. 3E). These results indicate that intestinal-specific *Nr1i2* deletion is insufficient to reproduce the chow-fed lean and muscular phenotype of whole-body *Nr1i2* knockout mice, pointing to the involvement of systemic or multi-organ mechanisms.

**Figure 3:**
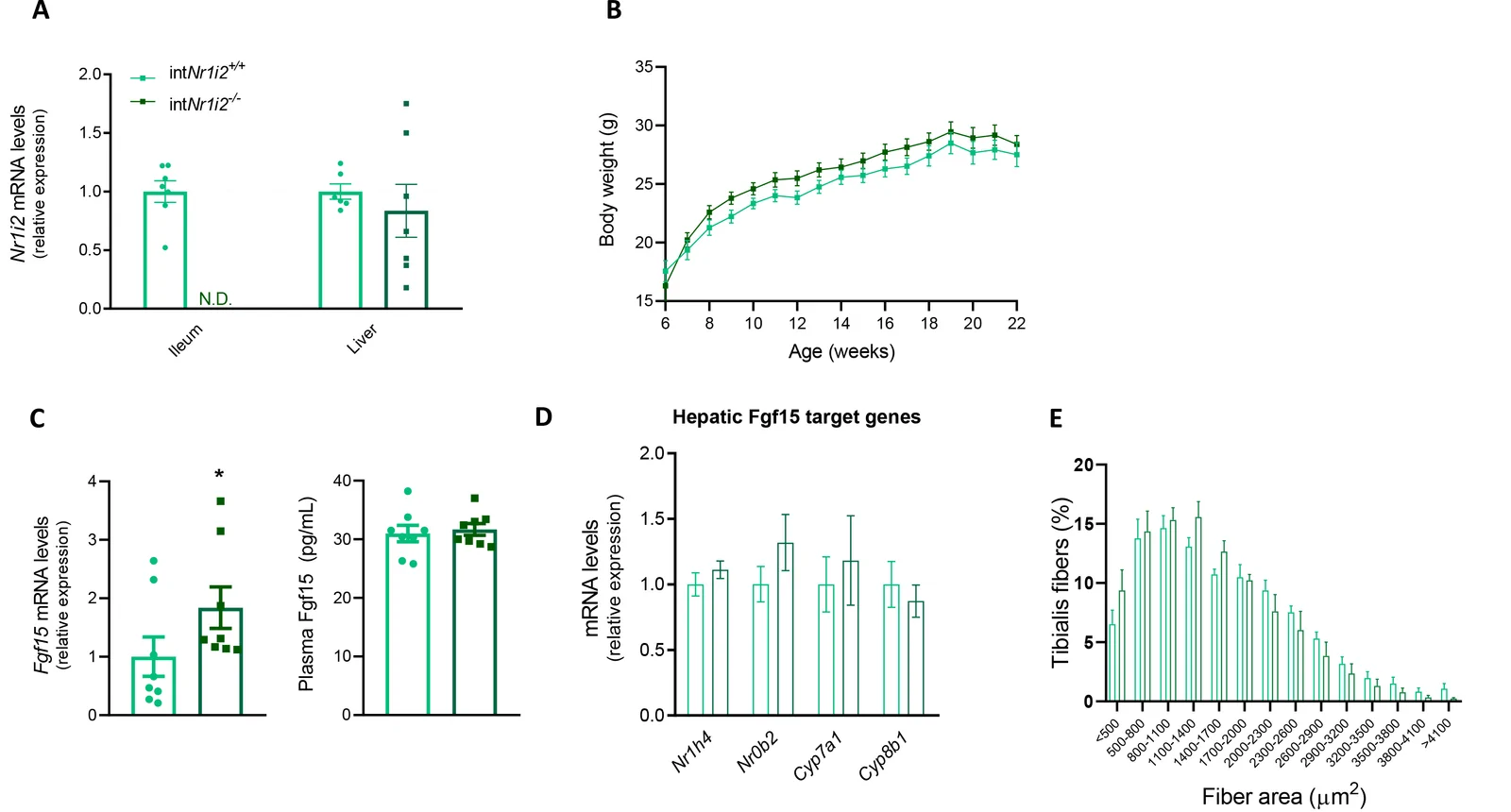
**Targeted intestinal *Nr1i2* deletion does not recapitulate the systemic effects observed in whole-body knockouts.** (A) Relative gene expression of *Nr1i2* in ileum and liver samples from intestinal-specific *Nr1i2* knock-out mice (*intNr1i2*^*−/−*^) and their wild-type littermates (*intNr1i2*^*+/+*^) mice. (B) Longitudinal changes in body weight (g) from 6 to 22 weeks of age. (C) *Fgf1*5 mRNA levels measured in the ileum and concentrations of FGF15 (pg/mL) measured by ELISA in plasma of *intNr1i2*^*+/+*^ and *intNr1i2*^*−/−*^mice fed a chow diet at 22 weeks of age following a 4-hour fast. (D) Relative gene expression of *Nr1h4*, *Nr0b2*, *Cyp7a1* and *Cyp8b1* in the liver of *intNr1i2*^*+/+*^ and *intNr1i2*^*−/−*^ mice. Gene expression data are presented as fold changes relative to gene expression levels in the control group (*intNr1i2*^*+/+*^ mice). (E) Fiber distribution (as %) based on area (in μm^2^) in the tibialis anterior muscles of *intNr1i2*^*+/+*^ and *intNr1i2*^*−/−*^ mice. Data are presented as mean ± SEM. ∗p < 0.05. N.D. indicates not detectable. *intNr1i2*^*+/+*^ mice are represented in light green, and *intNr1i2*^*−/−*^ mice in dark green. Group sizes ranged from n = 6 to 8 mice per group, depending on the parameter analyzed.

### *3.4* *Nr1i2*^*−/−*^ mice show elevated levels of conjugated bile acids in the small intestine

Given their central role in regulating FGF15/19 production through the intestinal FXR pathway [16,28], bile acids (BAs) were investigated as potential contributors to the elevated Fgf15 levels in *Nr1i2*^*−/−*^ mice. BA profiling was therefore performed in ileum samples. Total ileal BA levels were significantly increased in *Nr1i2*^*−/−*^ mice, driven primarily by elevated taurine-conjugated BAs. These changes were not observed in int*Nr1i2*^*−/−*^ mice, further supporting that intestinal-specific *Nr1i2* deletion does not reproduce the whole-body knockout phenotype (Figure 4A). Analysis of individual BA species revealed increased levels of both primary and secondary BAs in the ileum of *Nr1i2*^*−/−*^ mice (Figure 4B–C), with taurocholic acid (TCA) and tauro-α/β-muricholic acids (TMCA) as the main quantitative contributors (Fig. 4B). Notably, tauro-hyodeoxycholic acid (THDCA), undetectable in control animals, became the most abundant secondary BA in *Nr1i2*^*−/−*^ ileum. Additionally, the 6α-hydroxylated species, hyocholic acid (HCA) and hyodeoxycholic acid (HDCA), were exclusively detected in ileum of *Nr1i2*^*−/−*^ mice (Fig. 4C). Several other BAs, including taurodeoxycholic acid (TDCA), tauroursodeoxycholic acid (TUDCA), and 7-sulfocholic acid (CA-7S) were also significantly increased compared with *Nr1i2*^*+/+*^ mice (Figure 4B–C). Thus, a profound remodeling of the taurine-conjugated BA pool characterized the ileum of *Nr1i2*^*−/−*^ mice. Importantly, this remodeling involved qualitative changes in specific BA species rather than a uniform increase in total BA levels.

**Figure 4:**
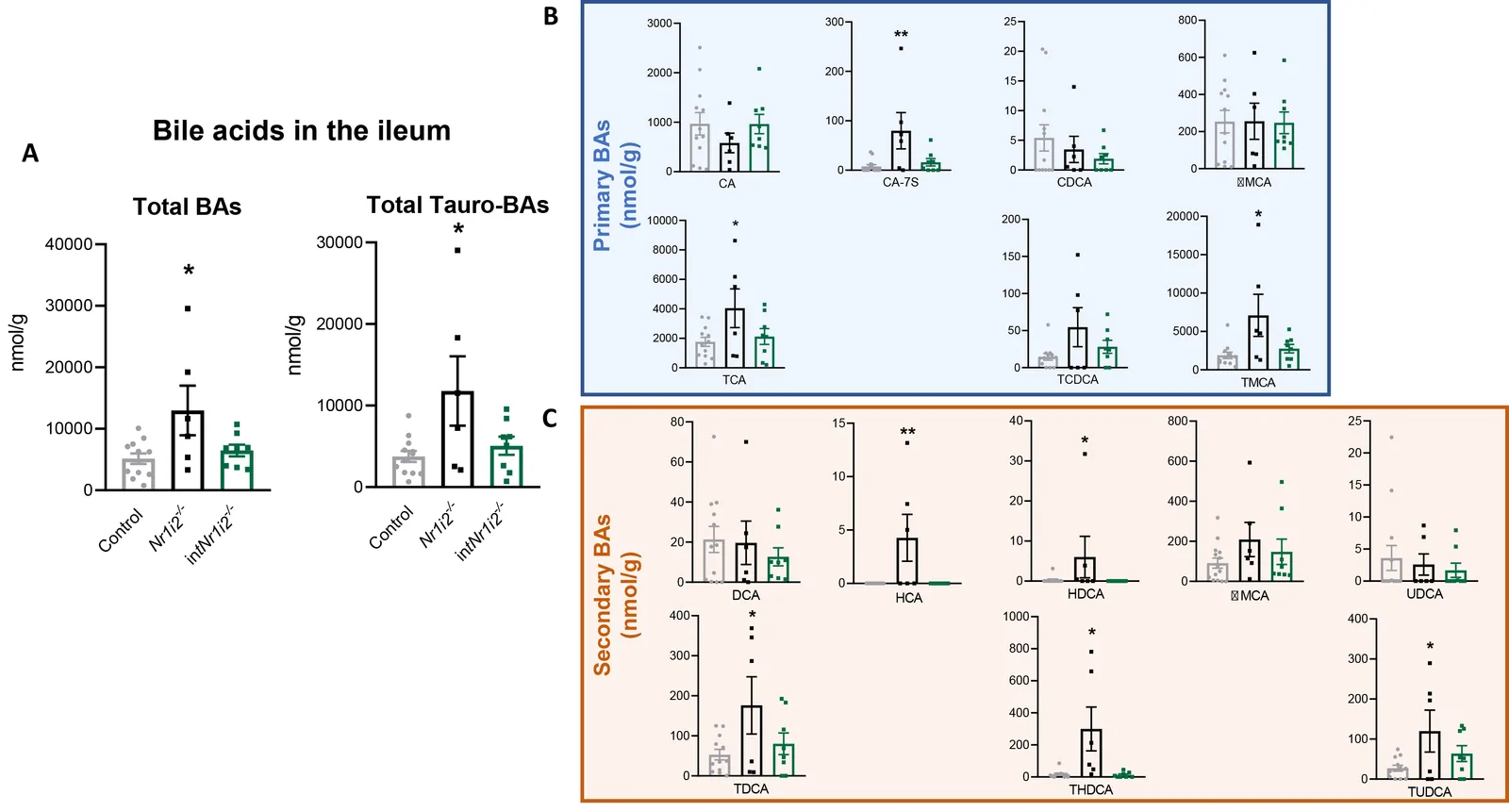
***Nr1i2*^*−/−*^mice display increased levels of taurine-conjugated bile acids in the ileum**. All graphs presented in this figure show bile acid concentrations measured in ileum lysates from mice in the wild-type (WT) groups (pooled data from *Nr1i2*^*+/+*^ and *intNr1i2*^*+/+*^ mice), whole-body *Nr1i2* knock-out (*Nr1i2*^*−/−*^), and intestinal-specific *Nr1i2* knock-out (*intNr1i2*^*−/−*^) groups. Bile acid concentrations are expressed in nmol/g of ileum tissue. (A) Total BAs: the sum of all measured bile acids, and total tauro-BAs: the sum of all measured tauro-conjugated bile acids. (B) Concentrations of all measured primary bile acids and their tauro-conjugated forms: CA (cholic acid), CA-7S (cholic acid 7-sulfate), CDCA (chenodeoxycholic acid), βMCA (beta-muricholic acid), TCA (taurocholic acid), TCDCA (taurochenodeoxycholic acid), and TMCA (tauromuricholic acid). (C) Concentrations of all measured secondary bile acids and their tauro-conjugated forms: DCA (deoxycholic acid), HCA (hyocholic acid), HDCA (hyodeoxycholic acid), ωMCA (omega-muricholic acid), UDCA (ursodeoxycholic acid), TDCA (taurodeoxycholic acid), THDCA (taurohyodeoxycholic acid), and TUDCA (tauroursodeoxycholic acid). Data are presented as mean ± SEM. ∗p < 0.05, ∗∗p < 0.01. *Nr1i2*^*+/+*^ and *intNr1i2*^*+/+*^ mice are represented in grey (control group), *Nr1i2*^*−/−*^ mice in black, and *intNr1i2*^*−/−*^ mice in dark green. Group sizes ranged from n = 6 to 11 mice per group, depending on the parameter analyzed.

### 3.5 Whole-body *Nr1i2* deletion reshapes hepatic bile acid metabolic pathways

Given that BAs are primarily synthesized and tauro-conjugated in the liver, hepatic BA composition was analyzed in *Nr1i2*^−/−^ and *Nr1i2*^+/+^ mice. Consistent with ileal findings, the hepatic BA pool was significantly increased in *Nr1i2*^*−/−*^ mice, with an approximately twofold enrichment in taurine-conjugated BAs (p = 0.055, Figure 5A). Among individual species, TMCA, TCA and TDCA levels were elevated (p = 0.053 for TCA, Figure 5B–C). Notably, THDCA was also increased in the liver of *Nr1i2*^*−/−*^ mice, paralleling ileal accumulation (Fig. 5C). Because BA accumulation can be associated with cholestatic liver injury, we assessed plasma alkaline phosphatase (ALP), a biochemical marker commonly elevated in cholestasis. Plasma ALP levels were not increased in *Nr1i2*^*−/−*^ mice compared with *Nr1i2*^*+/+*^ controls (Supp. Fig. 6A) suggesting that BA remodeling in chow-fed *Nr1i2*^*−/−*^ mice occurs without evidence of cholestatic injury. Since cholesterol is the precursor for bile acid synthesis, we next assessed whether hepatic bile acid remodeling was associated with altered cholesterol homeostasis. *Nr1i2*^*−/−*^ mice displayed reduced plasma cholesterol but increased hepatic cholesterol content compared with *Nr1i2*^*+/+*^ (Supp. Fig. 6B). In parallel, hepatic expression of key genes involved in cholesterol biosynthesis including *Srebf2*, *Hmgcs1* and *Hmgcr*, were not significantly modified in *Nr1i2*^*−/−*^ mice (Supp. Fig. 6C).

**Figure 5:**
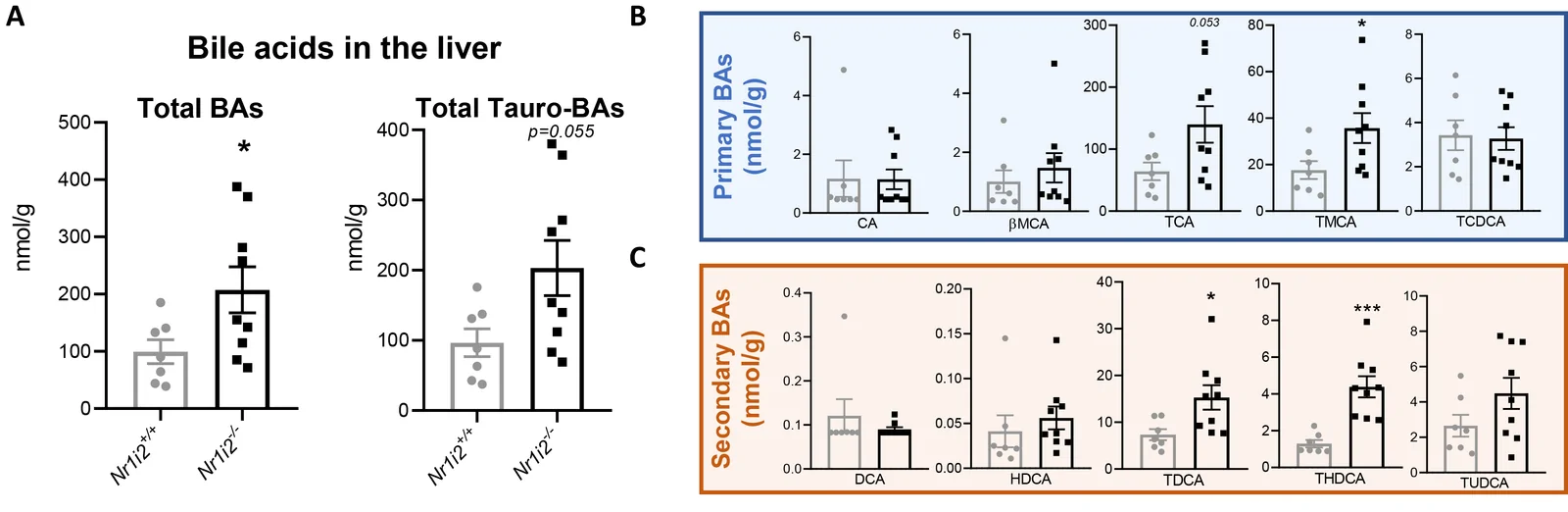
***Nr1i2*^*−/−*^mice display increased levels of taurine-conjugated bile acids in the liver.** All graphs display bile acid concentrations measured in liver lysates from *Nr1i2*^*+/+*^ and *Nr1i2*^*−/−*^ mice. Bile acid concentrations are expressed in nmol/g of liver tissue. (A) Total BAs: the sum of all measured bile acids, and total tauro-BAs: the sum of all measured tauro-conjugated bile acids. (B) Concentrations of all measured primary bile acids and their tauro-conjugated forms: CA (cholic acid), βMCA (beta-muricholic acid), TCA (taurocholic acid), TMCA (tauromuricholic acid) and TCDCA (taurochenodeoxycholic acid). (C) Concentrations of all measured secondary bile acids and their tauro-conjugated forms: DCA (deoxycholic acid), HDCA (hyodeoxycholic acid), TDCA (taurodeoxycholic acid), THDCA (taurohyodeoxycholic acid), and TUDCA (tauroursodeoxycholic acid). Data are presented as mean ± SEM. ∗p < 0.05, ∗∗∗p < 0.001. *Nr1i2*^*+/+*^ mice are represented in grey, and *Nr1i2*^*−/−*^ mice in black. Group sizes ranged from n = 7 to 9 mice per group, depending on the parameter analyzed.

We then examined hepatic gene expression related to BA synthesis, transport, and tauro-conjugation. Key enzymes of the alternative (acidic) BA synthesis pathway, including *Cyp27a1* and *Cyp7b1* were upregulated in *Nr1i2*^*−/−*^ mice (Figure 6A). *Cyp2c70*, which converts chenodeoxycholic acid (CDCA) to α/β-muricholic acids (MCA) [29] was also increased, while *Mafg* (musculoaponeurotic fibrosarcoma homolog g), a transcriptional repressor of BA synthesis [30], was significantly downregulated, supporting enhanced BA production. Regarding BA transport, expression of the sodium/bile acid cotransporter *Ntcp* (*Slc10a1*) and the bile salt export pump *Bsep* (*Abcb11*) was significantly increased in *Nr1i2*^*−/−*^ mice (Fig. 6A). Of note, these changes occurred without modification of hepatic *Nr1h4* mRNA expression (Fig. 2D).

**Figure 6:**
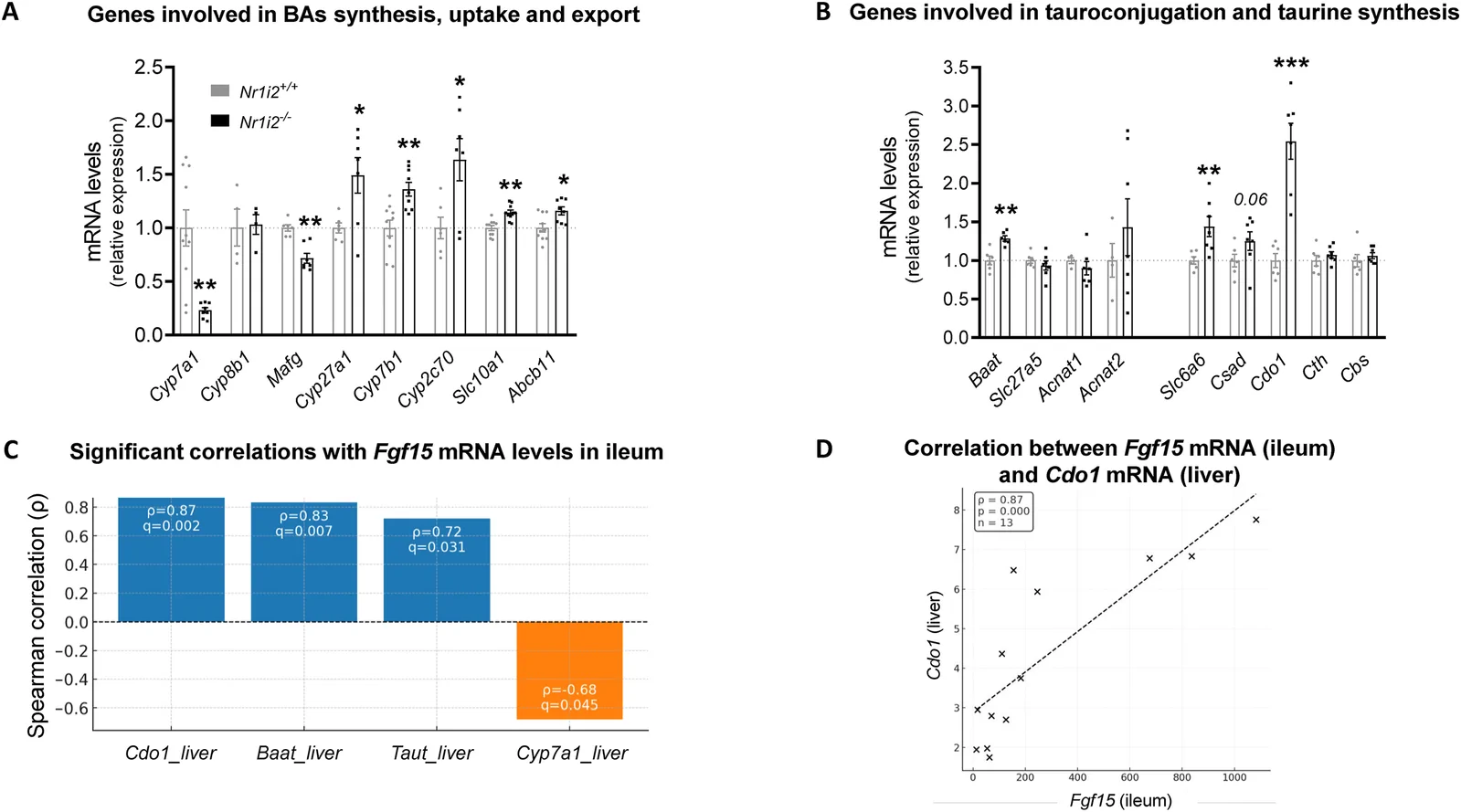
***Nr1i2*^*−/−*^deficiency remodels hepatic bile acid metabolism and links hepatic *Cdo1* expression to ileal *Fgf15* induction.** (A) Relative expression of genes involved in bile acid synthesis, uptake and export in the liver of *Nr1i2*^*+/+*^ and *Nr1i2*^*−/−*^ mice. Genes measured include *Cyp7a1*, *Cyp8b1*, *Mafg*, *Cyp27a1*, *Cyp7b1*, *Cyp2c70*, *Slc10a1*, and *Abcb11*. (B) Relative expression of genes associated with tauroconjugation and taurine synthesis in the liver of *Nr1i2*^*+/+*^ and *Nr1i2*^*−/−*^ mice. Genes analyzed include *Baat*, *Slc27a5*, *Acnat1*, *Acnat2*, *Slc6a6*, *Csad*, *Cdo1*, *Cth*, and *Cbs*. Gene expression data are presented as fold changes relative to gene expression levels in the control group (*Nr1i2*^*+/+*^ mice). (C) Spearman correlations between ileal *Fgf15* expression and hepatic gene expression. Only genes significantly correlated after multiple-testing correction (FDR <0.05, Benjamini–Hochberg method) are shown. Bars indicate correlation coefficients (ρ), with positive correlations in blue and negative correlations in orange; each bar is annotated with its corresponding ρ value and adjusted q-value. (D) Scatterplot showing the Spearman correlation between ileal *Fgf1*5 mRNA levels and hepatic *Cdo1* mRNA levels. Data are presented as mean ± SEM. ∗p < 0.05, ∗∗p < 0.01, ∗∗∗p < 0.001. *Nr1i2*^*+/+*^ mice are represented in grey, and *Nr1i2*^*−/−*^ mice in black. Group sizes ranged from n = 5 to 9 mice per group, depending on the parameter analyzed.

Concerning taurine conjugation (Fig. 6B), expression of the conjugating enzyme *Baat* (bile-acid-CoA:amino acid N-acyltransferase), was modestly but significantly increased in *Nr1i2*^*−/−*^, whereas *Slc27a5* (bile acid-CoA ligase), *Acnat1* and *Acnat2* (acyl-CoA:amino acid N-acyltransferases 1 and 2) remained unchanged. Genes involved in taurine uptake and biosynthesis, including *Taut* (taurine transporter, *Slc6a6*) and *Cdo1* (cysteine dioxygenase type 1) were upregulated while *Csad* (cysteine sulfinic acid decarboxylase) showed a trend toward significance (p = 0.06); *Cbs* (cystathionine beta-synthase) and *Cth* (cystathionine gamma-lyase), were unchanged (Fig. 6B). Together, these data suggest enhanced hepatic taurine availability, which, combined with increased *Baat* expression, supports elevated tauro-conjugation of BAs in *Nr1i2*^*−/−*^ mice.

To identify molecular pathways linking *Fgf15* induction to hepatic BA metabolism, we performed correlation analyses between ileal *Fgf1*5 mRNA levels and hepatic expression of genes involved in BA synthesis and conjugation. The analyses identified *Cdo1* as the hepatic transcript most strongly associated with ileal *Fgf15* expression (Figure 6C–D). To determine whether increased hepatic *Cdo1* expression resulted from elevated Fgf15 signaling rather than from Pxr deletion per se, *Cdo1* mRNA levels were measured in mice treated with human recombinant FGF19 [31] and in HepG2 cells exposed to FGF19 (Supp. Fig. 7A). In both models, *Cdo1* expression remained unchanged. To further assess whether CDO1 could be regulated by PXR activity in hepatocytes, HepG2 cells were treated with the PXR antagonist SPA70, alone or in combination with the PXR agonist rifampicin. As expected, SPA70 blunted rifampicin-induced *CYP3A4* expression, confirming effective pharmacological modulation of PXR activity (Supp. Fig. 7B). In contrast, *CDO1* expression was not significantly altered by either rifampicin or SPA70, alone or in combination (Supp. Fig. 7B). Moreover, *Cdo1* expression was increased in several extrahepatic tissues of *Nr1i2*^*−/−*^ mice (Supp. Fig. 7C), but was not altered in the ileum of intestinal-specific *Nr1i2*^*−/−*^ mice (Supp. Fig. 7D). These findings suggest that Cdo1 upregulation reflects a broader systemic response to *Nr1i2* deletion rather than a direct effect of Fgf15 or a direct hepatocyte-autonomous consequence of PXR loss.

### 3.6 Tauro-conjugated and 6α-hydroxylated bile acids stimulate FGF15/19 expression *in vitro*

To further evaluate the contribution of tauro-conjugated BAs to FXR-FGF15/19 pathway regulation, we used HT29 human intestinal epithelial cells, a human intestinal epithelial cell line commonly used to study FXR-dependent FGF19 responses [32]. Tauro-BAs increased in the ileum of *Nr1i2*^*−/−*^ mice (TCA, TDCA, THDCA, TβMCA and TUDCA) were tested for their ability to modulate FGF19 mRNA expression and secretion (Figure 7A–C). None of the tauro-BAs alone induced *FGF1*9 mRNA to levels comparable to the FXR agonist GW4064, although TCA and TDCA showed a trend (p = 0.11). When combined with GW4064, TCA and THDCA significantly potentiated FXR induced *FGF19* up-regulation (Fig. 7B). In contrast, FGF19 secretion was increased by TCA and TDCA when administered alone, and co-treatment with GW4064 further enhanced its secretion (TDCA + GW4064: 2-fold and THDCA + GW4064:1.8-fold; Fig. 7C). Similar but less prounonced effects on FGF19 secretion were observed with TCA and TUDCA, TβMCA did not change GW4064-induced FGF19 secretion (Fig. 7C).

**Figure 7:**
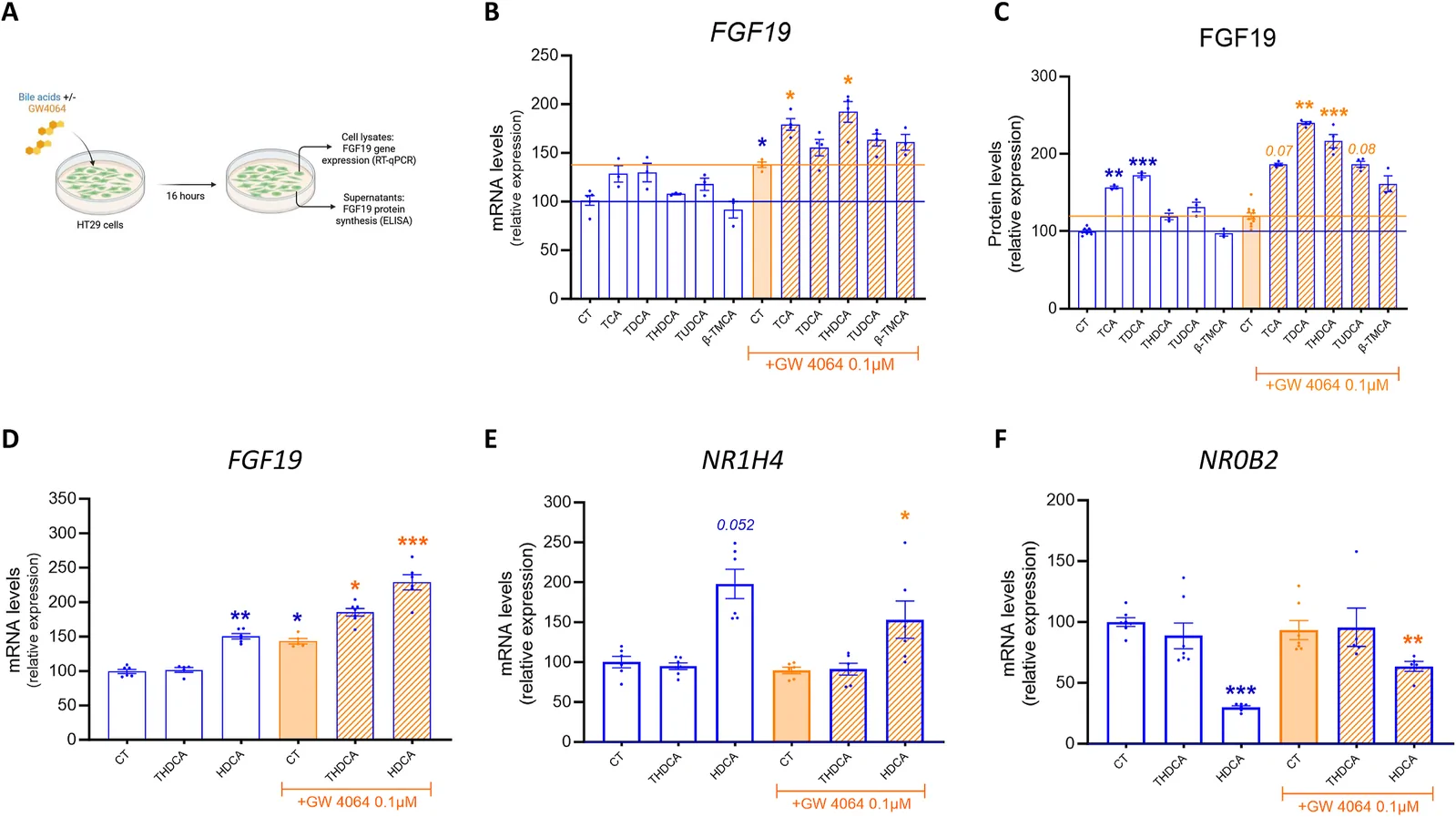
**THDCA and HDCA differentially modulate FGF19 synthesis in HT29 cells.** (A) Experimental design. (B) *FGF19* gene and (C) FGF19 protein secretion in cell supernatants in HT29 cells treated overnight with different bile acids (100 μM) tested either individually or in combination with GW4064 (0.1 μM). The bile acids tested include TCA (taurocholic acid), TDCA (taurodeoxycholic acid), THDCA (taurohyodeoxycholic acid), TUDCA (tauroursodeoxycholic acid) and β-TMCA (bêta-tauromuricholic acid). Gene expressions of (D) *FGF19* (E) *NR1H4* and (F) *NR0B2* in HT29 cells treated overnight with THDCA or HDCA (individually at 100 μM) or in combination with GW4064 (0.1 μM). All data presented in this figure are expressed as a percentage of the control group (CT), which is culture medium + DMSO. Blue asterisks indicate statistically significant differences between the control group (CT – medium + DMSO) and bile acid-only groups, while orange asterisks indicate statistically significant differences between the CT-GW4064 and bile acid + GW4064 combination groups. Results are shown as mean ± SEM. ∗p < 0.05, ∗∗p < 0.01, ∗∗∗p < 0.001. The control group (culture medium + DMSO) and bile acids tested individually are shown in dark blue, the GW4064 control is represented in orange, and the bile acid + GW4064 combinations are shown in dark blue with orange stripes. Group sizes ranged from n = 3 to 7, depending on the parameter analyzed.

Because THDCA was the most strongly increased BA species *in vivo*, we next compared the effects of THDCA and its unconjugated form HDCA on FXR signaling. HDCA alone significantly induced *FGF19* expression, whereas both BAs potentiated GW4064-induced *FGF19* expression, with HDCA exerting a stronger effect (Fig. 7D). Unlike THDCA, HDCA also induced *NR1H4* (encoding FXR, Fig. 7E) and repressed *NR0B2* (encoding SHP), a canonical FXR target (Fig. 7F), indicating distinct mechanisms of FXR modulation by 6α-hydroxylated BAs.

To validate these findings in a more physiological context, mouse ileal organoids were used (Figure 8A). HDCA significantly induced *Fgf15* expression, and GW4064 co-treatment further amplified this effect, recapitulating HT29 results (Fig. 8B). These effects were more modest with THDCA (Fig. 8B). As in HT29 cells, HDCA increased *Nr1h4* expression, whereas THDCA had no effect (Fig. 8C). HDCA also reduced *Nr0b2* expression, although this did not reach significance (p = 0.11, Fig. 8D). Together, these data collected from two different experimental settings indicate that HDCA and THDCA induce FGF15/19 via distinct FXR-related mechanisms.

**Figure 8:**
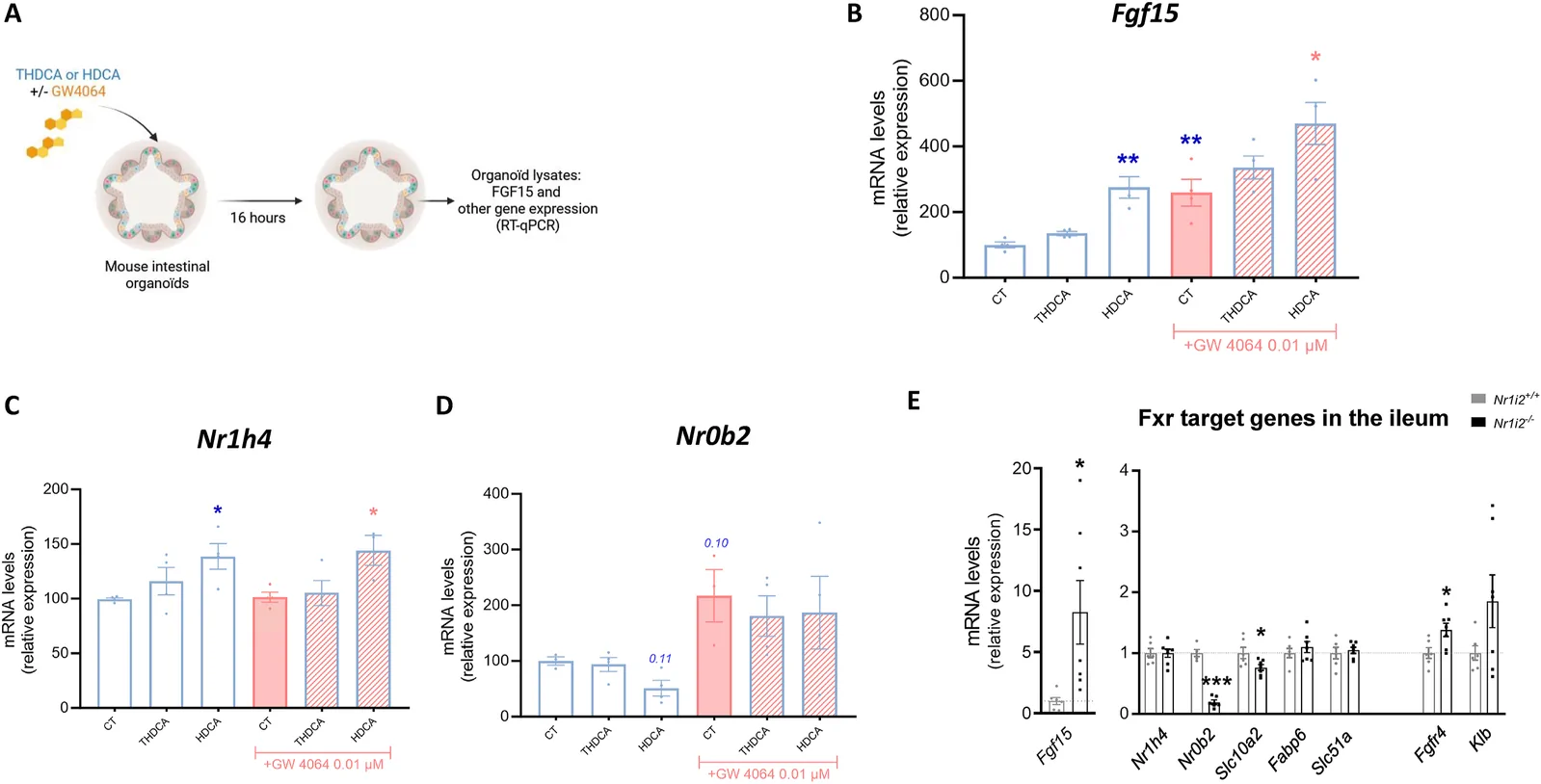
**THDCA and HDCA modulate the FGF15 pathway and may contribute to intestinal changes in *Nr1i2*^*−/−*^mice**. (A) Experimental design. Gene expressions of (B) *Fgf15* (C) *Nr1h4* and (D) *Nr0b2* in mouse ileal organoids treated overnight with THDCA or HDCA (individually at 100 μM) or in combination with GW4064 (0.01 μM). All data are expressed as a percentage of the control group (CT), which is culture medium + DMSO. Blue asterisks indicate statistically significant differences between the control group (CT – medium + DMSO) and bile acid-only groups, while light red asterisks indicate statistically significant differences between the CT-GW4064 and bile acid + GW4064 combination groups. The control group (culture medium + DMSO) and bile acids tested individually are shown in light blue, the GW4064 control is represented in red, and the bile acid + GW4064 combinations are shown in light blue with light red stripes. (E) Relative expression of Fxr target genes in the ileum of *Nr1i2*^*+/+*^ and *Nr1i2*^*−/−*^ mice including *Fgf15*, *Nr1h4*, *Nr0b2*, *Slc10a2*, *Fabp6*, *Slc51a, Fgfr4* and *Klb.* Gene expression data are presented as fold changes relative to gene expression levels in the control group (*Nr1i2*^*+/+*^ mice). *Nr1i2*^*+/+*^ mice are represented in grey, and *Nr1i2*^*−/−*^ mice in black. Results are shown as mean ± SEM. ∗p < 0.05, ∗∗p < 0.01, ∗∗∗p < 0.001. For organoid experiments, group sizes ranged from n = 3 to 7. For mouse experiments, group sizes ranged from n = 5 to 7 mice per group.

These findings prompted us to analyse the ileal FXR-related gene expression in *Nr1i2*^*−/−*^ mice (Fig. 8E). While *Fgf15* expression was strongly induced, the expression of other FXR-related genes - namely the apical sodium-dependent bile acid transporter (ASBT, encoded by *Slc10a2*) and *Nr0b2* - was significantly reduced, particularly for *Nr0b2*. The expressions of the bile acid-binding protein (*Fabp6*), the organic solute transporter alpha (OSTα, encoded by *Slc51a*) and FXR (*Nr1h4*) were unchanged (Fig. 8E). This dissociation does not support broad transcriptional activation of canonical ileal FXR target genes, but rather suggests selective regulation of the pathway, consistent with the *in vitro* bile acid responses. We also assessed whether increased ileal *Fgf15* expression was associated with changes in the Fgf15 receptor machinery. Ileal *Fgfr4* expression was significantly increased in *Nr1i2*^*−/−*^ mice, whereas *Klb* expression was not significantly modified, despite a tendency for higher mean expression in *Nr1i2*^*−/−*^ mice (Fig. 8E). These data suggest that *Nr1i2* deletion may modulate one component of the local Fgf15 receptor machinery, without demonstrating functional autocrine Fgf15 signaling in the ileum. Altogether, our findings support a role for increased 6α-hydroxylated and taurine-conjugated BAs in the selective upregulation of ileal Fgf15 in *Nr1i2*^*−/−*^ mice, together with possible modulation of local ileal FGF15/19 responsiveness.

## 4 Discussion

In the present study, we show that whole-body loss of Pxr promotes a lean phenotype accompanied by increased skeletal muscle mass in mice maintained on a standard chow diet. These findings extend previous studies performed in models of diet-induced obesity by showing that *Nr1i2* deficiency is sufficient to promote a lean phenotype even in the absence of obesogenic stress. Chow-fed *Nr1i2*^*−/−*^ mice exhibited reduced adiposity, improved lipid and glucose parameters, and increased energy expenditure despite increased food intake, indicating a sustained shift in energy balance independent of obesogenic stress. This phenotype was not associated with BAT activation or WAT browning but characterized by increased skeletal muscle mass and a shift toward larger muscle fibers, highlighting skeletal muscle remodeling as a major contributor to the systemic metabolic phenotype of Pxr-deficient mice.

A previous study reported increased intestinal *Fgf15* expression in HFD-fed Pxr-deficient mice [25] and we previously showed that FGF19 acts as a trophic factor for skeletal muscle in rodents [19,20,33]. Here, we extend these observations to chow-fed *Nr1i2*^*−/−*^ mice, in which robust ileal *Fgf15* induction was accompanied by elevated circulating Fgf15 levels. Despite the known technical limitations of Fgf15 quantification [34], these changes, together with the repression of hepatic *Cyp7a1,* are consistent with enhanced endocrine Fgf15 bioactivity *in vivo*. However, *Cyp7a1* repression cannot be attributed exclusively to Fgf15 signaling, since hepatic bile acid/FXR-dependent mechanisms may also contribute. In particular, unchanged hepatic *Nr1h4* mRNA expression does not exclude altered FXR activation, which may depend on ligand availability, post-translational regulation or nuclear localization. Thus, while our data support increased ileum-to-liver Fgf15 signaling, a contribution of hepatic FXR activation to *Cyp7a1* repression and bile acid pathway remodeling cannot be excluded.

We further assessed whether *Nr1i2* deletion altered local ileal expression of the Fgf15 receptor machinery. Ileal *Fgfr4* expression was increased, whereas *Klb* was not significantly modified, suggesting modulation of one receptor component without broad upregulation of the complete receptor complex. Therefore, the main functional evidence of enhanced Fgf15 bioactivity remains the endocrine ileum-to-liver response, although local ileal Fgf15 responsiveness may eventually also be affected. At the skeletal muscle level, *Nr1i2*^*−/−*^ mice showed increased expression of *Dio2*, *Ucp3*, *Gys1* and *Myod1* in the soleus, whereas *Fgfr4*, *Klb*, *Ucp2*, *Fbxo32* and *Trim63* were unchanged. These findings indicate that the increased soleus muscle mass and larger myofiber size observed in *Nr1i2*^*−/−*^ mice are associated with a transcriptional signature suggestive of enhanced energy/oxidative metabolism, increased glycogen storage capacity and myogenic remodeling without broad transcriptional upregulation of the Fgf15 receptor machinery or modulation of classical atrophy-related genes. Given the established role of FGF15/19 in regulating skeletal muscle mass and myotube hypertrophy [19,20,33], these data support the possible contribution of increased Fgf15 plasma levels to skeletal muscle remodeling and elevated energy expenditure in chow-fed *Nr1i2*^*−/−*^ mice, while the direct causality remains to be established.

To elucidate how *Nr1i2* ablation regulates Fgf15 production, we examined the role of intestinal epithelial Pxr, as Fgf15 is produced almost exclusively by the ileum [16]. However, intestinal-specific *Nr1i2* deletion failed to reproduce the lean phenotype, skeletal muscle remodeling, or increased Fgf15 bioactivity observed in whole-body *Nr1i2*^*−/−*^ mice, indicating that *Fgf15* induction is not driven by the loss of Pxr only in the intestinal epithelium.

We further investigated bile acids as Fgf15 regulators and found that whole-body, but not intestinal-specific, Pxr deficiency was associated with accumulation of tauro-conjugated and secondary 6α-hydroxylated bile acids in the distal small intestine, together with a shift from classical to alternative hepatic bile acid synthesis. These findings support a role for Pxr in regulating bile acid pool composition, which may influence enteroendocrine signaling. In line with cholesterol being the precursor for bile acid synthesis, *Nr1i2*^*−/−*^ mice also displayed reduced plasma cholesterol and increased hepatic cholesterol content, although this was not associated with any induction of cholesterol biosynthesis genes. Thus, Pxr deficiency appears to alter cholesterol metabolism, but the bile acid remodeling observed in these mice is unlikely to reflect a generalized activation of hepatic cholesterol biosynthesis.

Analysis of the intestinal bile acid handling revealed a modest reduction of *Slc10a2* (Asbt) expression, while other Fxr-regulated transporters remained unchanged. *Nr1i2*^*−/−*^ mice also displayed dissociation between *Fgf15* induction and the expression of other canonical ileal Fxr target genes such as *Nr0b2* [35], suggesting a selective or non-canonical regulation of intestinal Fxr-related signaling [36]. Consistently, *in vitro* experiments in human intestinal cells and mouse ileal organoids demonstrated that specific bile acid species differentially regulate *FGF19* and *NR0B2* expression, supporting the concept that bile acid composition, rather than total bile acid levels, can selectively modulate FGF15/19 endocrine signaling.

Among the bile acids enriched in *Nr1i2*^*−/−*^ mice, 6α-hydroxylated species have been associated with improved metabolic health in rodents and humans [12,[37], [38], [39]]. Their emergence in the ileum, together with their ability to stimulate *Fgf15*, suggests that they may contribute to the metabolic and muscular phenotype observed in *Nr1i2*^*−/−*^ mice. Beyond FXR-related pathways, these bile acids may also activate the G protein–coupled bile acid receptor TGR5, promoting GLP-1 production [12,37]. Consistently, ileal *Gcg* expression was increased in *Nr1i2*^*−/−*^ mice (Supp. Fig. 8), suggesting that Glp-1 signaling may represent an additional pathway linking bile acid remodeling to metabolic benefits in Pxr-deficient mice under chow diet.

Collectively, our findings identify Pxr/*Nr1i2* as a regulator of body composition and skeletal muscle mass under physiological dietary conditions. By showing for the first time that *Nr1i2* deficiency promotes a lean phenotype with increased skeletal muscle mass in chow-fed mice, this study broadens the metabolic role of PXR beyond obesogenic settings and provides mechanistic clues linking the lean phenotype to bile acid remodeling and increased Fgf15 signaling.

Several limitations should be acknowledged. Although intestinal-specific Nr1i2 deletion did not reproduce the whole-body knockout phenotype, contributions from other Pxr-expressing tissues, particularly the liver, cannot be excluded given its role in bile acid synthesis and the shift from classical to alternative bile acid pathways observed in *Nr1i2*^*−/−*^ mice. Future studies using liver-specific Nr1i2 deletion or specific liver re-expression of PXR in globally deficient mice will be required to establish its contribution to the phenotype. Additionally, as the study relies predominantly on mRNA expression data, future Western blotting or immunohistochemical analyses will be needed to confirm that the reported transcriptional changes are reflected at the protein level. Another limitation is the fact that this study was conducted in adult male mice only, and potential sex-specific effects of Nr1i2 deficiency cannot be excluded, particularly since PXR knockout was previously shown to mitigate weight gain and hepatic metabolic dysregulation in female mice under long-term high-fat diet conditions [40]. Future studies will be required to determine whether the bile acid remodeling, increased Fgf15 signaling, and skeletal muscle hypertrophy identified here are conserved in females. Also, whole-body and intestinal-specific *Nr1i2* knockout models were also maintained on distinct genetic backgrounds, which may have influenced the magnitude or expression of metabolic phenotypes. Additionally, gut microbiota composition was not directly assessed. Because microbiota-dependent transformation of bile acids may have contributed to the observed bile acid remodeling, particularly the accumulation of secondary and 6α-hydroxylated bile acids, future studies integrating microbiota profiling will be required. Finally, species-specific differences in bile acid metabolism and FGF15/19 signaling should be considered when extrapolating these findings to human physiology.

## CRediT authorship contribution statement

**Bérengère Benoit:** Writing – original draft, Visualization, Validation, Project administration, Investigation, Formal analysis, Conceptualization. **Audrey Jalabert:** Investigation. **Emmanuelle Meugnier:** Writing – review & editing, Investigation, Formal analysis. **Stéphanie Chanon:** Investigation. **Aurélie Vieille-Marchiset:** Investigation. **Alice Beau:** Investigation. **Sandra Pesenti:** Investigation. **Emmanuelle Loizon:** Investigation. **Oriane Vitalis:** Investigation. **Nadia Bendridi:** Investigation. **Margaux Nawrot:** Writing – review & editing, Methodology, Investigation. **Kassem Makki:** Writing – review & editing, Formal analysis. **Brigitte Le Magueresse-Battistoni:** Writing – review & editing, Formal analysis. **Jennifer Rieusset:** Writing – review & editing, Investigation. **Marie-Caroline Michalski:** Writing – review & editing. **Dominique Rainteau:** Writing – review & editing, Investigation, Formal analysis. **Karim Chikh:** Writing – review & editing, Investigation. **Hubert Vidal:** Writing – original draft, Supervision, Project administration, Methodology, Funding acquisition, Formal analysis, Conceptualization, Validation. **Jérôme Ruzzin:** Writing – original draft, Validation, Supervision, Project administration, Methodology, Investigation, Funding acquisition, Formal analysis, Conceptualization.

## Declaration of generative Ai and ai-assisted Technologies in the manuscript preparation process

During the preparation of this work, the author used ChatGPT in order to improve the readability and language of the manuscript. After using this tool, the author reviewed and edited the content as needed and takes full responsibility for the content of the published article.

## Funding sources

This work was supported by the 10.13039/501100005416Research Council of Norway (NFR), INSERM, and the 10.13039/501100001665French National Research Agency (ANR; grant ANR-19-CE14-0017-01). B.B. was supported by a postdoctoral fellowship from the NFR Environmental Exposures and 10.13039/100018696Health Outcomes program, followed by an 10.13039/501100001665ANR postdoctoral grant. J.R. was supported by the FRIPRO Young Research Talents program.

## Declaration of competing interest

The authors declare no competing interests.

## Data Availability

Data will be made available on request.
