# Supplementary material for: Pregnane X receptor deletion induces a lean phenotype in chow-fed mice through a liver–ileum–muscle axis involving 6α-hydroxylated bile acids and Fgf15
Source: Mol Metab. 2026 Sep 19;113:102442. doi: 10.1016/j.molmet.2026.102442 (PMC13651484; doi:10.1016/j.molmet.2026.102442)
Supplement: Supplementary file 1 [file mmc1.docx]

**Pregnane X receptor deletion induces a lean phenotype in chow-fed mice
through a liver–ileum–muscle axis involving 6α-hydroxylated bile acids and Fgf15**

Bérengère Benoit,**^a,*^** Audrey Jalabert,**^a^** Emmanuelle Meugnier,**^a^** Stéphanie Chanon,**^a^** Aurélie Vieille-Marchiset,**^a^** Alice Beau,**^a^** Sandra Pesenti,**^a^** Emmanuelle Loizon,**^a^** Oriane Vitalis,**^a,b^** Nadia Bendridi,**^a^** Margaux Nawrot,**^a^** Kassem Makki,**^a^** Brigitte Le Magueresse-Battistoni,**^a^** Jennifer Rieusset,**^a^** Marie-Caroline Michalski,**^a^** Dominique Rainteau,**^c^** Karim Chikh,**^a,b^** Hubert Vidal,**^a,#^** and Jérôme Ruzzin **^d,e,#^**

**Supplementary methods:**

### **Statistical analysis of body weight evolution over time**

Body weight was measured longitudinally in *Nr1i2^-/-^* and *Nr1i2^+/+^* mice over several consecutive weeks or months under high-fat diet or chow conditions. Repeated measurements were obtained from the same animals across time.

Body weight data were analyzed using **linear mixed-effects models** to account for repeated measurements within individual mice. Genotype (WT vs KO), time, and their interaction were included as fixed effects. Mouse identity was included as a random effect (random intercept) to account for inter-individual variability.

Time was treated as a continuous variable to assess overall body weight trajectories and genotype-specific weight gain over time. The significance of fixed effects, including the genotype × time interaction, was assessed using Wald tests.

To identify time points at which body weight differed between genotypes, post-hoc pairwise comparisons were performed at each individual week. For these analyses, time was treated as a categorical variable, and estimated marginal means were calculated for each genotype at each time point. Pairwise contrasts between *Nr1i2^-/-^* and *Nr1i2^+/+^* mice were computed for each week.

P-values from post-hoc analyses were adjusted for multiple comparisons using the **Benjamini–Hochberg false discovery rate (FDR)** procedure. An FDR-adjusted p-value < 0.05 was considered statistically significant.

All statistical analyses were performed using the R statistical software. Linear mixed-effects models were fitted using the **lme4** package, and post-hoc analyses were conducted using the **emmeans** package.

### **Statistical analysis of muscle fiber size distribution**

Muscle fiber size distribution was analyzed in soleus and tibialis anterior muscles from *Nr1i2^-/-^* and *Nr1i2^+/+^* mice. For each mouse, muscle fibers were classified into predefined size bins, and the proportion of fibers within each bin was calculated.

Fiber size distribution data were analyzed using a **generalized linear mixed-effects modeling approach** to account for the repeated-measures structure of the data, with multiple size bins measured within each individual mouse. Genotype (WT vs KO), fiber size bin, and their interaction were included as fixed effects. Mouse identity was included as a random effect (random intercept) to account for inter-individual variability.

Proportion data were modeled on the logit scale, and the significance of fixed effects was assessed using likelihood ratio tests. A significant genotype × fiber size bin interaction was interpreted as evidence of a difference in the overall fiber size distribution profile between genotypes.

Post-hoc pairwise comparisons between *Nr1i2^-/-^* and *Nr1i2^+/+^* mice were performed for each fiber size bin using model-derived estimated marginal means. P-values were adjusted for multiple comparisons using the **Benjamini–Hochberg false discovery rate (FDR)** procedure. An FDR-adjusted p-value < 0.05 was considered statistically significant.

All statistical analyses were performed using the R statistical environment. Mixed-effects models were fitted using the **lme4** package, and post-hoc analyses were conducted using the **emmeans** package.

**Plasma ALP measurement**

Plasma alkaline phosphatase (ALP) concentrations were measured using a Mouse Alkaline Phosphatase ELISA Kit (ab285274, Abcam, UK), according to the manufacturer's instructions.

**Plasma and hepatic cholesterol measurements**

Total cholesterol concentrations were measured in plasma samples and liver lysates using the Cholesterol PAP kit (Biolabo, France) according to the manufacturer's instructions. For hepatic cholesterol determination, frozen liver samples were homogenized in lysis buffer, and cholesterol concentrations were measured in the resulting lysates. Absorbance was recorded at 500 nm and cholesterol concentrations were calculated using the supplied calibrator. Plasma cholesterol concentrations were expressed as gl/L, whereas hepatic cholesterol concentrations were normalized to tissue weight and expressed as µg/mg liver.

**PXR modulation in HepG2 cells**

HepG2 cells were cultured in standard growth medium and seeded in 24-well plates at approximately 180,000 cells per well. Cells were allowed to reach 70–85% confluence before treatment. Cells were then incubated for 24 h with the PXR antagonist SPA70 (2.5 µM or 5 µM), the PXR agonist rifampicin (10 µM), or a combination of both compounds. Vehicle-treated cells (0.1% DMSO) served as controls. At the end of the treatment period, total RNA was extracted and reverse-transcribed into cDNA. Gene expression was quantified by RT-qPCR using specific primers for *CYP3A4* and *CDO1*. *CYP3A4* expression was used as a positive control to confirm effective pharmacological modulation of PXR activity. Experiments were performed in two independent biological replicates with technical duplicates for each condition.

**Supplementary Figures**

**Supplementary Fig. 1**

mRNA expression levels of *Nr1i2* (in arbitrary units) in the ileum, liver, and soleus muscle of *Nr1i2^+/+^* and *Nr1i2^-/-^* mice. N.D. indicates not detectable. Results are expressed as means ± SEM. n=4 mice/group.

**Supplementary Fig. 2**

Relative gene expression of *Fgfr4*, *Klb*, *Dio2*, *Ucp2*, *Ucp3*, *Gys1*, *Myod1*, *Fbxo32* and *Trim63* in the soleus muscle of mice. Results are expressed as means ± SEM. * p*<0.05*. *Nr1i2^+/+^* mice are represented in grey, and *Nr1i2^-/-^* mice are represented in black. n=4 to 6 individuals for the *Nr1i2^-/-^* group and the *Nr1i2^+/+^* group.

******

iWAT

eWAT

**Supplementary Fig. 3**

Parameters related to non-shivering thermogenesis and hepatic lipid metabolism in *Nr1i2^+/+^* and *Nr1i2^-/-^* mice fed a chow diet for 22 weeks. (**A**) Relative gene expression of *Gpbar1*, *Pgc1α*, *Pgc1β*, *Ucp1*, *Ucp2*, and *Ucp3* in the brown adipose tissue of mice. (**B**) Relative gene expression of *Ucp1*, *Ucp2*, and *Tmem26* in the epididymal white adipose tissue (eWAT) and inguinal white adipose tissue (iWAT) of mice. (**C**) Rectal temperature in °C of the mice. (**D**) Relative gene expression of *Cd36*, *Srebp1c*, *Fasn*, *Dgat2*, *Pparα*, *Pgc1α*, *Pdk4*, *Cpt1a*, and *Ucp2* in the livers of the mice. Results are expressed as means ± SEM. * p*<0.05*; ** *p<0.01*. *Nr1i2^+/+^* mice are represented in grey, and *Nr1i2^-/-^* mice are represented in black. n=4 to 6 individuals for the *Nr1i2^-/-^* group and the *Nr1i2^+/+^* group.

iWAT

BAT

eWAT

**Supplementary Fig. 4**

Panels A to D depict data from *Nr1i2^+/+^* and *Nr1i2^-/-^* mice fed a chow diet from birth to 17 months of age. (A) Longitudinal changes in body weight (g) from birth to 17 months of age and food intake (food intake was measured during the 18^th^ month of the protocol). (B) Weights of epididymal white adipose tissue (eWAT), inguinal white adipose tissue (iWAT), and brown adipose tissue (BAT) collected from 17-month-old mice. (C) Weights of soleus, tibialis anterior, and gastrocnemius muscles collected from 17-month-old mice. (D) Plasma insulin, triglycerides, and blood glucose levels (parameters measured at 17 months following 4 hours of fasting). Data are presented as mean ± SEM. *p<0.05, **p<0.01, ***p<0.001. n = 5 individuals per group.

BAT

iWAT

eWAT

**Supplementary Fig. 5**

Phenotyping of *Nr1i2^-/-^* mice fed a high-fat diet. (**A**) Evolution of body weight in mice (from 6 to 22 weeks of age) and food intake expressed in grams of food consumed per day (recorded during the 12^th^ of the diet). (**B**) Weight of epididymal white adipose tissue (eWAT), inguinal white adipose tissue (iWAT), and brown adipose tissue (BAT) collected from 22-week-old HFD mice. (**C**) Weights of soleus, tibialis anterior, and gastrocnemius muscles collected from 22-week-old HFD mice. (**D**) Glucose tolerance test: blood glucose levels (in mmol/L) over time (in min) and graphical representation of the area under the curve calculated from the test data. (**E**) Insulin sensitivity test: evolution of blood glucose levels (expressed as a percentage of fasting blood glucose, i.e., at t0) over time (in min) and graphical representation of the area under the curve calculated from the test data. (**F**) Blood glucose levels (in mmol/L), insulin levels (in ng/mL), and triglyceride levels (in g/L) after fasting. (**G**) Triglyceride content in the liver (in µg/mg) with representative Oil Red O staining images of *Nr1i2^+/+^* and *Nr1i2^-/-^* mice. Triacylglycerol levels were determined in frozen liver samples from using high-performance thin-layer chromatography, and fixed (paraffin-embedded) liver sections were stained, 60x magnification, scale bar = 25 µm. Results are expressed as means ± SEM. * *p<0.05*; ** *p<0.01*; and ****p<0.001*. n=6 to 16 individuals for the *Nr1i2^-/-^* group and n=8 to 18 individuals for the *Nr1i2^+/+^* group.

C

**Supplementary Fig. 6**

(A) Plasma ALP levels (in pg/mL), (B) Plasma cholesterol levels (in g/L) and hepatic cholesterol levels (in µg/mg liver). (C) Relative gene expression of *Srebf2*, *Hmgcs1* and *Hmgcr* in the liver of mice. Results are expressed as means ± SEM. ** *p<0.01*. *Nr1i2^+/+^* mice are represented in grey, and *Nr1i2^-/-^* mice are represented in black. n=6 to 11 individuals for the *Nr1i2^-/-^* group and the *Nr1i2^+/+^* group.

**Supplementary Fig. 7**

(A) Relative *Cdo1* expression in livers of mice treated for 21 days with human recombinant FGF19 (0.1 mg/kg; dark blue) or vehicle (PBS–0.1% BSA; light blue), and confluent HepG2 (human hepatocellular carcinoma) cells treated overnight with 5 ng/mL recombinant human FGF19 (dark blue with dots) or vehicle (light blue with dots). Data are expressed as percentage of the respective vehicle controls. (B) Relative expression of ***CYP3A4*** and ***CDO1*** in HepG2 cells treated with the PXR antagonist SPA70 (2.5 or 5 µM), the PXR agonist rifampicin (10 µM), or their combination. *CYP3A4* was used as a positive control for PXR modulation. Data are presented as mean ± SEM. (C) Relative *Cdo1* expression across multiple tissues (liver, epididymal white adipose tissue, ileum, soleus, gastrocnemius) in *Nr1i2^⁺/⁺^* (grey) and *Nr1i2^⁻/⁻^* (black) mice. Data are expressed as fold change relative to *Nr1i2^⁺/⁺^* controls. (D) Relative *Cdo1* expression in ileum and liver samples from intestine-specific *intNr1i2^⁺/⁺^* (light green) and *intNr1i2^⁻/⁻^* (dark green) mice. Data are expressed as fold change relative to *intNr1i2^⁺/⁺^* controls. ** *p<0.01*; and ****p<0.001*.

**Supplementary Fig. 8**

Relative expression of *Gpbar1* and *Gcg* in the ileum of *Nr1i2^+/+^*(grey) and *Nr1i2^-/-^* (black) mice. Data are presented as mean ± SEM. **p<0.01.

**Supplementary Tables**

**Supplementary Table 1:** Mouse primer sequences.

| **Primer sequences for RT-qPCR analysis of mouse tissues** | | |
| --- | --- | --- |
| Target Gene | Forward Primer 5'-3' | Reverse Primer 5'-3' |
| *Abcb11* | GCAGTCACACCCATCTACTC | TGGGAAGCATCTGTAGCAAG |
| *Acnat1* | AGACCTGCCTGAGAAACTGC | TTATGCAGACCGTGGCAATC |
| *Acnat2* | AGACCTGCCTGAGAAACCAC | GGCCCATTTATGCAGACTGT |
| *Baat* | CTGGCTTACTGGAACTATGA | TCTTCATTACTGGGTACAGG |
| *Cbs* | TGTGGTCATCCTGCCTGACT | GACAGGCTCAGCTCTTGAAC |
| *Cd36* | GTCCTGGCTGTGTTTGGAGG | AAGATCCAAAACTGTCTGTA |
| *Cdo1* | TTCGATACATGCCACGCCTT | TTGGTGGGCCCTAGTTGTTC |
| *Cpt1a* | ACGTATGAGGCTTCCATGAC | GGTGAGTCGACTGCCAGATA |
| *Csad* | CCAGGACGTGTTTGGGATTGT | ACCAGTCTTGACACTGTAGTGA |
| *Cth* | TCGGGATCAATGACACACTG | CGTATGCTCCGTAATGATCT |
| *Cyp24a1* | TGGAGACGACCGCAAACAG | AGGCAGCACGCTCTGGATT |
| *Cyp27a1* | TCTGGCTACCTGCACTTC | ACCACACCAGTCACTTCC |
| *Cyp27b1* | TTGGCAAACGGAGCTGCATC | TTGATGCTCCTCTCAGGGAC |
| *Cyp2b10* | TTCTGCCCTTCTCAACAGGA | CCTTAGGAGCAACATGGCTT |
| *Cyp2c70* | GTGCATTTGTTCCCAGTGAGTC | GCTTCATGCCCAGGTATACAGT |
| *Cyp3a11* | ACGCCTCTCCTTGCTGTCACC | TTGCCTTCTGCCTCAAGTAC |
| *Cyp7a1* | CAATGAAAGCAGCCTCTGAAG | AAAAGTCAAAGGGTCTGGG |
| *Cyp7b1* | CCCATACTTAGTATCTGACATACC | TGGAATGGTGTTTGCTAGAG |
| *Cyp8b1* | ATGTCCTGAAGATGGCCAGC | GGCATGCTGTAGTGGTGGAT |
| *Dgat2* | TGGGTCCAGAAGAAGTTCCAGAAGTA | ACCTCAGTCTCTGGAAGGCCAAAT |
| *Fabp6* | TACGATGAGTTCATGAAGCG | CGGTAGCCTTGAACTTCTTG |
| *Fasn* | CTGTGCTTGCAGCTTACTGG | ACGGTGTCCTCAGAGTTGTG |
| *Fgf15* | GTCGCTCTGAAGACGATTGC | GCTTCATGGATCTGTACTGG |
| *Fgfr4* | CAACTCCATCGGCCTTTCCT | CAGAACCAGTGAGCCTGATA |

**Supplementary Table 2:** Mouse primer sequences.

| **Primer sequences for RT-qPCR analysis of mouse tissues** | | |
| --- | --- | --- |
| Target Gene | Forward Primer 5'-3' | Reverse Primer 5'-3' |
| *Gcg* | GCCGTGCCCAAGATTTTGTG | CCTTTCACCAGCCAAGCAATG |
| *Gpbar1* | GTCAGTCTTGGCCTATGAGC | AGTGCTGGGGCCTGGATTG |
| *Il10* | CAGGGCCCTTTGCTATGGTG | CGGCTGGGGGATGACAGTAG |
| *Mafg* | CAAGGCCTTAAAGGTGAAGC | CCAGCTCCTCCTTCTGTGT |
| *Nr1h4* | GCAGACCTGTTGGAAGAAAG | GTCTGTCTGGAGAGAGGATG |
| *Nr1i2* | AGGAGGAGTATGTGCTGATG | CTTCAGGAACAGGAACCTGTG |
| *Nrob2* | CTGCCTGGAGTCTTTCTGGA | GTGCCTGGAATGTTCTTGAG |
| *Pdk4* | AGTGTGCAAAGATGCTCTGC | AGAGCATGTGGTGAAGGTGTG |
| *Pgc1α* | TCCTCTGACCCCAGAGTCAC | CTTGGTTGGCTTTATGAGGAGG |
| *Pgc1β* | GGAAGCGGCGGGAAAAGGCC | GCTGTCAAAATCCATGGCTTC |
| *Pparα* | GAGAATCCACGAAGCCTAC | GAAGAATCGGACCTCTGCCTC |
| *Scl10a1* | CTTAAGGACAAGGTGCCCTAC | GTTGCCCACATTGATGACAG |
| *Slc10a2* | GGATCTCAACCTGGTGTTCA | ATCAAATGATGGCCTGGAGT |
| *Slc27a5* | GAGCTTGTACAGTTCGACAT | CAGTGTTGAAGTACAGGTCT |
| *Slc51a* | AGAACACCCTTTGCCCCATC | CAAAGCCTTCCACCATGACC |
| *Slc6a6* | GACTACTATGCAGCTAGTGG | GTAGGTCAGGGGTACATACT |
| *Srebp1c* | ACGGAGCCATGGATTGCACA | AAGGGTGCAGGTGTCACCTT |
| *Sult2a1* | CAGATGAGCTGGATCTCGTCC | GTGATTCTTCCAGTCCCCAA |
| *Tbp* | TGGTGTGCACAGGAGCCAAG | TTCACATCACAGCTCCCCAC |
| *Tmem26* | TAGGACTCCACCAAACACTC | ACCAGTTTCAACTGGACTGC |
| *Trpv6* | GATGGGTGACACTCACTGGA | GATGCGTTGTCTGTTGAGAT |
| *Ucp1* | ACCACAGAAAGCTTGTCAAC | AAGAGCTGACAGTAAATGGC |
| *Ucp2* | CCTGAAAGCCAACCTCATGAC | AGGATCCCAAGCGGAGAAAGG |
| *Ucp3* | CCTACAGAACCATCGCCAGG | ACCGGGGAGGCCACCACTGT |
| *Vdr* | GAATGTGCCTCGGATCTGTGG | ATGCGGCAATCTCCATTGAAG |

**Supplementary Table 3:** Human primer sequences.

| **Primer sequences for RT-qPCR analysis of cell culture samples (HT29 and HepG2 cell lines)** | | |
| --- | --- | --- |
| Target Gene | Forward Primer 5'-3' | Reverse Primer 5'-3' |
| *CDO1* | GAGTCTTGAGGGAAAACCAG | GATCAAAGGCATGGCATGTA |
| *CYP3A4* | ACCGAGTGGATTTCCTTCAG | AGTTTCTGCTGGACATCAGG |
| *FGF19* | CAGTTTGCTGGAGATCAAGG | AGCATGGGCAGGAAATGAG |
| *NR1H4* | GGCAACCAATCATGTACAGG | CGAAGGAACATAGCTTCAACC |
| *NROB2* | CATCCTCTTCAACCCCGATGT | AGGCTGGTCGGAATGGACTT |
| *TBP* | AGACCATTGCACTTCGTGCC | CCTGTGCACACCATTTTCCC |
